# Myopia prevalence, refractive status and uncorrected myopia among primary and secondary school students in Germany

**DOI:** 10.3389/fmed.2024.1483069

**Published:** 2024-12-12

**Authors:** Astrid Hönekopp, Lisa-Marie Tommes, Philipp Doebler, Sarah Weigelt

**Affiliations:** ^1^Research Unit Vision, Visual Impairments and Blindness, Department of Rehabilitation Sciences, TU Dortmund University, Dortmund, Germany; ^2^Research Unit Statistical Methods in the Social Sciences, Department of Statistics, TU Dortmund University, Dortmund, Germany

**Keywords:** myopia prevalence, school myopia, autorefraction, spherical equivalent, refractive error

## Abstract

**Introduction:**

The increasing prevalence of myopia worldwide is problematic because myopia can result in severe secondary pathologies, and is associated with considerable financial burden. With plenty of prevalence data available for some regions, current data for Europe remain sparse. Yet, information on myopia prevalence and associations is essential for monitoring, preventive and interventive purposes. Likewise, uncorrected refractive errors are also critical, as they can, e.g., affect educational outcomes, making information on uncorrected myopia valuable for diagnostics and health education.

**Methods:**

We performed non-cycloplegic autorefraction on two samples in Germany. The younger sample included 489 primary school students (grades 3–4, mean age: 9.30 ± 0.78 years), the older sample 1,032 secondary school students (grades 8–10, mean age 14.99 ± 1.12 years). These samples mark the limits of the age range during which school myopia usually emerges.

**Results:**

Myopia (spherical equivalent ≤ −0.75D) prevalence was 8.4% in the younger sample and 19.5% in the older sample. The prevalence was generally higher in higher grade levels, with the most notable difference between grades 8 and 9. Females were more myopic than males in all grades except grade 3, with the largest gender difference in grade 10. The older sample also exhibited a more myopic spherical equivalent than the younger sample. In the older sample, spherical equivalent was more myopic in females than in males, and in grade 9 and 10 participants more than in grade 8 participants. Rates of uncorrected myopia were extremely high: 51.2% in the younger sample and 43.3% in the older sample.

**Discussion:**

The obtained myopia prevalence rates are generally consistent with other European studies, as is the higher prevalence in female than male adolescents, accelerating with age. The high rates of uncorrected myopia warrant further investigation and should inform public health policies, including the implementation of regular refractive screenings.

## 1 Introduction

The global prevalence of myopia is increasing and has been estimated to become as high as 49.8% in 2050, if not for control interventions (1). In parts of East Asia, myopia prevalence has even reached alarmingly high rates of up to 90% in adolescents and young adults (2, 3), with high myopia being as high as 10–20% (3). In Europe, both myopia and high myopia prevalence are considerably lower than in (East) Asia (4, 5). Yet, increases in myopia prevalence have been observed in Europe as well (1). As myopia can cause substantial individual and public financial burden (6, 7), and high myopia is associated with an increased risk of severe secondary pathologies (8), this global increase in myopia prevalence requires immediate attention.

Unfortunately, current European data on myopia prevalence are rather sparse. In two recent reviews on myopia epidemiology in school children (9) and on epidemiological data on myopia from population-based studies published (4), only nine publications reporting myopia prevalence in Europe (geographical definition) were identified (4, 9). Therein, myopia prevalence rates of school-aged children and adolescents vary between 2.4% [6-year-olds; (10)] and 42.7% [10-19-year-olds; (11)]. High myopia prevalence rates were consistently low (0- < 2%) in the three publications reporting them (11–13). For Germany, a steady increase in myopia prevalence from 2.08% at age 3 to 25.87% at age 17 has been reported (14) – though again, data are sparse. To reduce this data gap, we investigated myopia epidemiology, including myopia prevalence, in school children in Germany.

With school age as a critical period for myopia development (15, 16), several underlying factors might be target points for prevention and intervention. School myopia (or juvenile myopia) has been described to appear between the ages of 9 and 11, and to then progress up to the late teenage years or early twenties (17). In a broader definition, school myopia is described to appear between the ages of 8 and 14 years, with potential further progression up to the age of approximately 30 (15). Less bright (outdoor) light exposure and more near work are considered important environmental factors driving this development (16). Furthermore, myopia prevalence and academic achievement are often linked in the literature, and while the direction of causality has been a matter of debate, current evidence strongly suggests that education plays a causal role in myopia development (5). Regarding gender, in white and East Asian populations, myopia prevalence differences have been found to appear around age 9 and become more pronounced thereafter, with a higher prevalence in girls than boys (18). Up-to-date information on myopia prevalence, its development during school age and associated factors such as these is important for monitoring purposes and to create interventions for prevention and diagnostics of myopia (progression).

In this regard, uncorrected refractive errors are a most relevant issue as they are problematic in many ways. Uncorrected refractive errors constitute the principal cause of visual impairments globally (19). In a recent meta-analysis, the global potential productivity loss associated with visual impairments due to uncorrected myopia has been estimated at USD $244 billion dollars, thereby substantially exceeding the cost of myopia correction (20). Uncorrected refractive errors have also been shown to affect children’s educational outcomes. For example, children who were provided free glasses upon failing visual acuity screenings (and having improved visual acuity with refraction) improved in mathematics test scores (21). The analysis’ effect size increased with increasing blackboard use during teaching, making it plausible for myopic children to have especially benefited from the provision of glasses, and underlining the impact of providing spectacles to myopic children (21). Thus, information on magnitude and associations of uncorrected myopia are important from a public health standpoint and may contribute to identifying relevant aspects regarding health education and diagnostic interventions.

Here, we performed non-cycloplegic autorefraction in two samples of school students in Germany. The chosen samples constitute the upper and lower limit of the age range in which school myopia usually first appears. We aimed at estimating the prevalence of myopia and uncorrected myopia among primary and secondary school students in Germany and analyzing potential sociodemographic predictors for refractive status.

## 2 Materials and methods

The study was approved by the local ethics board at TU Dortmund University and followed the tenets of the Declaration of Helsinki.

### 2.1 Participants

We recruited primary and secondary schools within the German federal state North Rhine-Westphalia, where children are usually 6 or 7 years old when they enroll in primary school (grades 1–4). After that, most students visit one of four types of secondary schools: The general secondary school (“Hauptschule,” grades 5–10) can be completed with the lower secondary school leaving certificate, the intermediate secondary school (“Realschule,” grades 5–10) with the secondary school certificate, and the grammar school (“Gymnasium,” grades 5–12 or 5–13) with the general qualification for university entrance (A-levels). Lastly, the comprehensive school (“Gesamtschule”) combines all aforementioned courses of education and school-leaving certificates. The school system slightly differs between German federal states, but is generally comparable.

For each of these types of school, we created randomized lists of all schools around a city in North Rhine-Westphalia, and contacted schools accordingly. We chose the first six consenting primary schools and the first consenting secondary school per type for participation. Since no grammar school from the initial list agreed to participation, we contacted another grammar school located just outside the originally determined area, which participated. Subsequently, we invited all students of grades 3 and 4 (primary schools) as well as grades 8, 9, and 10 (secondary schools) to participate via a multilingual letter distributed 2 weeks prior to the testing date(s). Therein, families were told to inform the school if they did not want their child to participate. We were able to conduct this opt-out procedure as the need for informed parental was waived by the ethics committee due to the non-invasiveness of our measurements and the immediate data anonymization (see below).

In the participating primary schools, 581 students were enrolled in grades 3 and 4, and thus eligible for our younger sample (3th- and 4th-grade primary school students; S1). Of those, 489 students participated. For our older sample (8th-, 9th-, and 10th-grade secondary school students; S2), 1,344 students were enrolled in the respective grades and thus eligible for participation, and 1,032 students participated. Most of the eligible non-participating students were not at school during the testing dates due to Covid-19 related quarantine. One participant in S1 and two participants in S2 were excluded from all analyses, because we were unable to obtain the relevant measurements. Thus, the final sample comprised of 488 participants (mean age: 9.30 ± 0.78 years) in S1 and 1,030 participants (mean age: 14.99 ± 1.12 years) in S2, and approximately reflected the lower and upper limits of the age range in which school myopia typically appears (15).

### 2.2 Study design

Data was collected at the schools between September and December 2021, i.e., during the first 4 months of the school year. To conduct all measurements, four trained experimenters visited each primary school for 1 day, and each secondary school for three consecutive days. In sum, 18 experimenters were involved in data collection. Participants completed the measurements individually, three at a time in the same room.

Upon participation, participants received a feedback card for their caregivers. All cards specified that we had measured refraction to detect ametropia. The other information varied based on our results: (1) If the participant had a visual aid, the family was reminded that regular visits to an ophthalmologist are advisable even in the absence of complaints. (2) If the participant had no visual aid and we detected no abnormalities, the family was informed about this and also told that regular visits to an ophthalmologist are advisable even in the absence of complaints. (3) If the participant had no visual aid and we detected abnormalities, the family was informed about this along with the recommendation to undergo an ophthalmological examination. Cards (2) and (3) also explicitly stated that our measurements were not medical diagnoses. We did not include refractive values in the feedback, nor did we prescribe refractive correction for participants ourselves.

### 2.3 Measurements

We measured non-cycloplegic refraction three times with one of three autorefractometers (2x model A12R with software 7.1.8.0, 1x model A09 with software 5.0.22.0; Plusoptix GmbH, Nürnberg, Germany) at a distance of 1 meter – i.e., each participant was measured with one of the three devices. The mean spherical equivalent refraction (sphere + 1/2 cylinder; SER) of these measurements was taken for analysis. If a participant wore glasses, we obtained their specifications by an auto lensmeter (model TL-3000C; Tomey Corporation, Nagoya, Japan). If a participant wore contact lenses during measurements, we obtained information about their kind from the participant.

We also recorded participant-reported gender (male/female/non-binary) and age in days, calculating the latter from the participant’s reported birth date for immediate anonymization.

Furthermore, we obtained each school’s social index level as a school-based measure of social burden (22) from the website of North Rhine-Westphalia’s ministry of education (23). The social index is a measure to identify the need for support of individual schools in North Rhine-Westphalia due to the students’ social composition. It is based on child and youth poverty as well as proportion of students with primarily non-German family language, who immigrated to Germany, and with special educational needs in learning, language, or emotional and social development. The social index is calculated on a scale from zero to 100 and each score is assigned to a social index level between one and nine, with one reflecting a low social burden (22).

### 2.4 Data analysis

Data analysis was conducted using R 4.4.1 (24) in RStudio 2021.9.0.351 (25) as well as the packages *psych* (26), *mgcv* (27), *MuMIn* (28), and *ggplot2* (29). The significance level was set at *α* = 0.05. If corrections were applied to calculations, e.g., for multiple comparisons, corrected *p*-values are reported.

#### 2.4.1 Data preparation

For four participants, we could only perform refractive measurements while they were wearing their glasses. We calculated their sphere and cylinder values by adding the respective glasses’ specifications to the measured values. Then, we calculated mean SER as we did for the other participants.

Furthermore, we were not able to perform all three refractive measurements with some participants. If data from only one (two) measurement(s) were available, we used these data to calculate mean SER. For the right eye, this was the case for four (nine) participants, and for the left eye, for five (nine) participants, respectively.

Nine participants wore contact lenses during measurements and were thus excluded from all SER analyses.

Lastly, the autorefractometers have a measurement range from -7D to +5D SER in 0.25D steps for sphere and cylinder (30, 31). We replaced participants’ values measured as “out of range” in the myopic (hyperopic) direction by −7.125D (+5.125D) SER as the next lower (higher) SER possible with the devices’ 0.25D steps for sphere and cylinder. For the right eye, this was the case for seven (seven) participants, and for the left eye, for eight (nine) participants, respectively.

We tested the comparability between the measurements of the two autorefractometer models used for data collection in a comparison study: We measured 58 additional participants three times with each autorefractometer and calculated mean SER for each eye for each device. Paired t-tests, corrected with Holm’s method for multiple comparisons (32), showed that the mean SER values obtained with the A09 device differed significantly from those of either A12R device (all *p*s < 0.001), while there was no difference between the A12R devices (both *p*s > 0.05). Visual inspection of LOWESS lines and Cronbach’s Alpha with the devices as “items” (both eyes: *α* = 0.99) indicated linear relationships between the mean SER values of all devices. Subsequently, we fitted generalized additive models including a smooth term to the comparison study data using the default generalized cross-validation to determine the degree of smoothness. The smooth used the usual wiggliness penalty of the second derivative, but no null space so that the F-test is a test of non-linearity. We predicted the mean SER of either A12R device for right and left eyes independently. As generalized cross-validation could potentially undersmooth, the same analysis was performed with the REML criterion as part of the sensitivity analysis. For all models, the smooth term was not significant (all *p*s > 0.05), again corrected for multiple comparisons using Holm’s method (32). Therefore, we assumed a linear relationship between measurements with the A09 device and measurements with the A12R devices, and pursued a linear transformation of the A09 data to obtain comparable data for all participants from the actual study, regardless of the autorefractometer they had been measured with. To this end, we averaged the mean SER of the A12R devices for each participant in the comparison study, and fitted linear models to predict this averaged mean SER of the A12R devices from the mean SER of the A09 device for each eye. Finally, we linearly transformed the data from the actual study that had been measured with the A09 device with the obtained regression coefficients for each eye. Detailed results of the analyses mentioned in this paragraph are presented in Supplementary Tables S1–S5 and Supplementary Figure S1. In all following analyses, we used the linearly transformed data of the A09 device along with the (non-transformed) data of the A12R devices.

We rechecked all calculations reported in the results section (1) with the complete data without linear transformation of the A09 device data and (2) with the data from the A12R devices only (see Supplementary Tables S6–S23 and Supplementary materials S1–S4). Despite a few quantitative and qualitative differences between the data analytic approaches, the overall results’ patterns did not change.

Since the SER of the right and left eye were well correlated (Spearman’s rho; *r*_s_ = 0.82, *p* < 0.001) and not significantly different from each other (*p* = 0.12), only data of the right eye are presented in the following.

#### 2.4.2 Myopia prevalence

We calculated myopia prevalence for S1 and S2 overall and for various subgroups. We defined myopia as SER ≤ −0.75D (14, 33, 34) to compensate for myopia overestimation due to the non-cycloplegic nature of the autorefraction measurements. Myopia prevalence values are reported for this cut-off, if not stated otherwise. Prevalence rates for the SER ≤ −0.5D myopia definition are also reported for comparability with other investigations (13, 35, 36). High myopia was defined as SER ≤ -6D (12, 35, 37).

For the myopia prevalence, we included all participants with usable SER data as well as participants without usable SER data, if we could derive the type of ametropia from their visual aid (i.e., measured or reported specifications). Usable SER data includes SER data obtained from successful autorefraction measurements from participants not wearing visual aids as well as successful autorefraction measurements over glasses, in which case we calculated the participants’ actual SER as described earlier. Non-usable SER data therefore entails unsuccessful autorefraction measurements due to measurement complications or autorefraction measurements over contact lenses, in which case we could not determine the actual, uncorrected SER. Measurement complications entailing the absence of SER data only occurred in participants with visual aids. For one participant in S1 and two participants in S2, no SER data exists due to such complications, and we also do not have any information on their visual aid specifications. Thus, these three participants were excluded from analysis, as has already been described with regard to the participant sample. Further two participants in S1 and 10 participants in S2 had no usable SER data, but we were able to derive their type of ametropia from their visual aids, and thus included them in the analyses on myopia prevalence.

For the prevalence estimation of high myopia, we only included participants with usable SER data since we could not always obtain detailed specifications of participants’ visual aid and visual aid specifications do not always fit the magnitude of a refractive error.

#### 2.4.3 Refractive status associations

An independent samples t-test was conducted to confirm SER differences between S1 and S2. Subsequently, we performed multiple linear regression analysis with SER as outcome for S1 and S2 separately, including grade and gender as predictors. Though age usually also predicts SER, we did not include both age and grade in the initial model as they are highly correlated (Spearman’s rho; overall: *r*_s_ = 0.91; S1: *r*_s_ = 0.62; S2: *r*_s_ = 0.76). We included grade based on the assumption that years of schooling may play a role in myopia development and because, e.g., Wang et al. (38) had found grade to predict myopia and vision impairment slightly better than age – which was also the case in our data for both S1 and S2. We subsequently applied the “all possible subsets” approach to test if there was a better regression model for either sample. Thereby, every possible predictor combination is run (automatically) to obtain the best combination of potential predictors – thus, this approach can be a useful screening to reduce the number of possible models (39). We tested age, gender, and grade as potential predictors and also included their interaction terms, constraining the inclusion of the latter in that they could only be included if the respective main terms already were. Both gender and grade were treated as categorical predictors.

We assessed the models obtained via the “all possible subsets” approach using adjusted *R^2^* and the Bayesian information criterion (BIC). Adjusted *R^2^* indicates the amount of variability of the dependent variable explained by a regression model. While *R^2^* always increases when more potential predictors are added to a model, irrespective of whether they add real predictive value, adjusted *R^2^* takes this potential overestimation into account and gives a more realistic estimation of the model’s performance (40). The BIC balances model fit and complexity. As models with more predictors always fit the data better than models with fewer ones, the BIC “penalizes” the addition of parameters to the model (41). In model selection, one aims maximizing *R^2^* and minimizing BIC.

In all regression analyses, we only included participants with available data for the considered predictors and the outcome. Furthermore, as there were only four non-binary participants, we only included males and females in these analyses. Post-hoc comparisons were conducted for significant predictors.

#### 2.4.4 Uncorrected myopia

To estimate the prevalence of participants with uncorrected myopia, we divided the number of myopic participants that reported absence of a visual aid by the number of myopic participants that reported either absence or presence of a visual aid. Thereby, we did not consider whether the specifications of participants’ visual aids matched our refraction measurements nor whether the visual aid had actually been prescribed for myopia correction. We additionally performed the estimation of uncorrected myopia prevalence with a more conservative SER ≤ -1D myopia cut-off.

Furthermore, we exploratively investigated potential associations between uncorrected myopia and the schools’ social index level in S1 by separately calculating the prevalence of uncorrected myopia for the three primary schools with the lowest and the highest social index level.

## 3 Results

In the following, results on myopia prevalence, associations with spherical equivalent, and the prevalence of uncorrected myopia will be presented. As described above, myopia was defined as SER ≤ −0.75D, and high myopia as SER ≤ -6D. Additionally, myopia prevalence rates are reported for the SER ≤ −0.5D cut-off.

The final sample included 488 participants in S1 (mean age: 9.30 ± 0.78 years; gender: 266 male, 219 female, 3 n/a) and 1,030 participants in S2 (mean age: 14.99 ± 1.12 years; gender: 571 male, 454 female, 4 non-binary, 1 n/a). The number of participants included in the individual analyses is stated throughout in the following.

### 3.1 Myopia prevalence

As expected, myopia prevalence was higher in the older than the younger sample: In our sample of children aged 9.30 ± 0.78 years (S1), myopia prevalence was 8.4%, while in our sample of children aged 14.99 ± 1.12 years (S2), myopia prevalence was 19.5%. High myopia was extremely low, affecting only two children (0.4%) S1 and eight children (0.7%) in S2, respectively, which aligned with our expectations as well.

Furthermore, and also unsurprisingly, myopia prevalence was higher in higher versus lower grades: In our younger sample (S1), myopia prevalence was 8.2% in grade 3 and 8.6% in grade 4, and in our older sample (S2), it was 11.6% in grade 8, 21.5% in grade 9, and 25.7% in grade 10, respectively. Accordingly, the prevalence difference was particularly notable between grades 8 and 9. High myopia only occurred in one participant per grade in S1. In S2, the relative frequency of high myopia slightly increased with increasing grade level, but again, extremely few participants were affected: one in grade 8, two in grade 9, and five in grade 10. Table 1 displays myopia and high myopia prevalence rates per sample and grade in detail.

**Table 1:** Myopia and high myopia prevalence in S1 and S2 overall and by grade.

	Myopia				High myopia
Sample	Age *M* (SD)	*N*	% ≤ −0.75D	% ≤ −0.5D	% ≤ −6.0D
S1	9.30 (0.78)	488	8.4	11.3	0.4
Grade 3	8.85 (0.73)	245	8.2	10.2	0.4
Grade 4	9.75 (0.53)	243	8.6	12.3	0.4
S2	14.99 (1.12)	1,030	19.5	28.8	0.7
Grade 8	13.98 (0.77)	346	11.6	18.2	0.3
Grade 9	15.04 (0.80)	349	21.5	30.1	0.6
Grade 10	15.97 (0.73)	335	25.7	38.5	1.2

Age and N are presented for the sample included in the myopia prevalence calculation. For the high myopia prevalence calculation, 2 (10) of these participants were excluded from S1 (S2) as described in Data Analysis. Thus, 486 participants (age: 9.29 ± 0.77 years) were included in the high myopia prevalence calculation for S1, and 1,020 participants (age: 14.98 ± 1.12 years) for S2.

We also discovered interesting results with regard to gender: As presented in Table 2, myopia prevalence for males was 7.5% and for females 9.6% in our younger sample (S1), while it was 14.9% for males and 24.9% for females in our older sample (S2). Thus, while myopia prevalence was comparable between genders in S1, many more females than males exhibited myopia in S2. High myopia occurred relatively more often in males than females in both samples (see Table 2). A higher prevalence of myopia in adolescent females than males is expected, but the gender differences in S2 are surprisingly large, as will be presented in the following.

**Table 2:** Myopia prevalence in S1 and S2 by gender.

	Myopia				High myopia
Sample	Age *M* (SD)	*N*	% ≤ −0.75D	% ≤ −0.5D	% ≤ −6.0D
S1					
Female	9.27 (0.77)	219	9.6	11.0	0.0
Male	9.33 (0.79)	266	7.5	11.7	0.8
S2					
Female	14.90 (1.05)	454	24.9	33.3	0.4
Male	15.05 (1.17)	571	14.9	25.0	0.9

Four participants with unknown gender and four non-binary participants were excluded from these calculations. Age and N are presented for the sample included in the myopia prevalence calculation. For the high myopia prevalence calculation, 2 (10) of these participants were excluded from S1 (S2) as described in Data Analysis. Thus, 483 participants (age: 9.30 ± 0.78 years) were included in the high myopia prevalence calculation for S1, and 1,015 participants (age: 14.98 ± 1.12 years) for S2.

**Figure 1:**
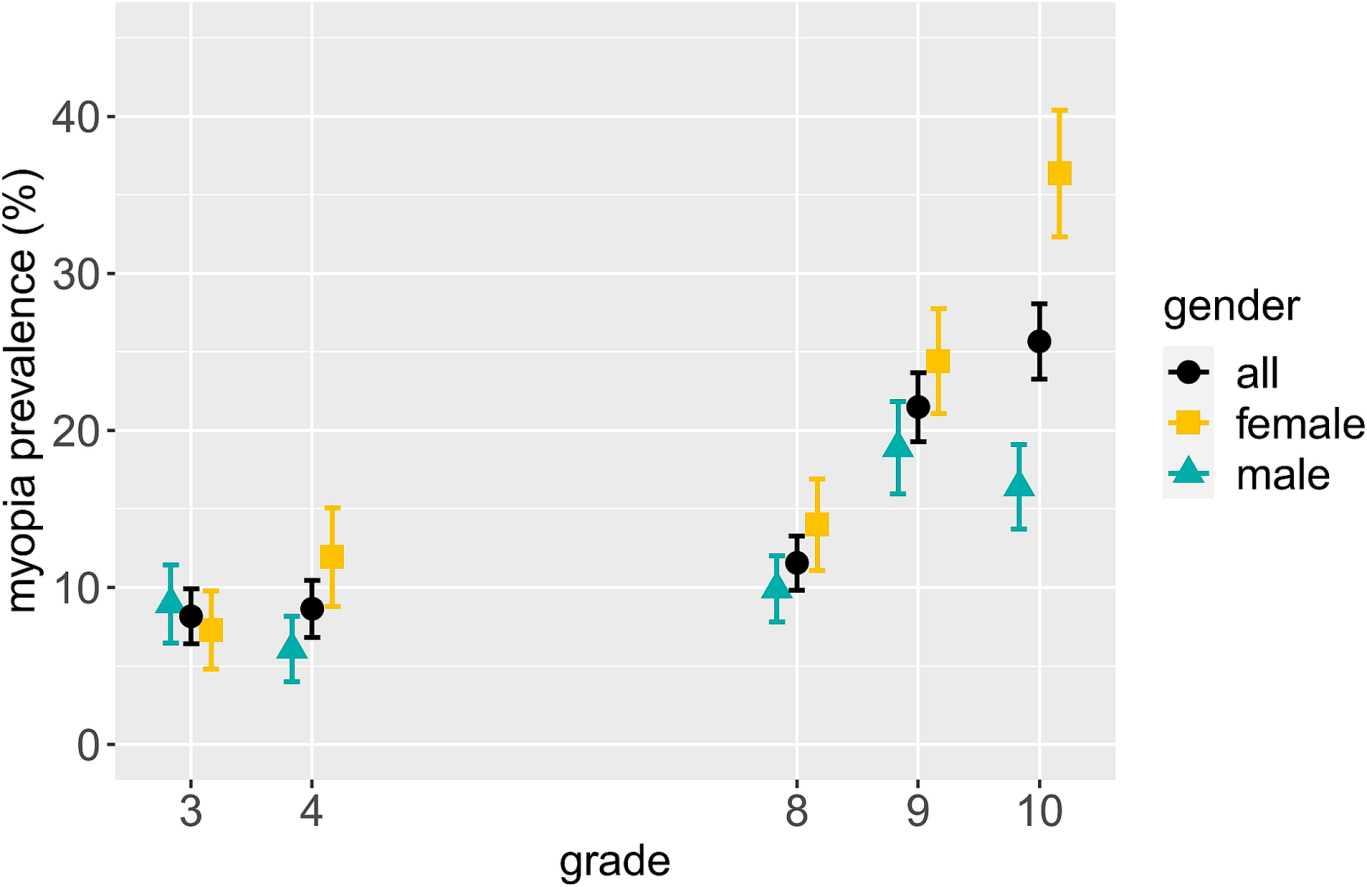
Myopia prevalence and standard error per gender by grade. The data for all genders include eight more participants than the data of males and females combined due to four non-binary participants and four participants with unknown gender.

The accelerating gender difference with increasing age can be seen even more clearly in individual grades: Not only was the myopia prevalence (overall and in individual grades) in females higher than in males in the older sample (S2), but the between-grade prevalence differences were also more pronounced in females than in males (see Figure 1): The prevalence difference between grade 8 and 9 is comparable for males (9.0%) and females (10.4%), but there is a 22.4% higher myopia prevalence for females in grade 10 than 8, while the prevalence of males in grade 10 is only 6.5% higher than of those in grade 8. Interestingly, while myopia prevalence was virtually similar between genders in grade 3 (1.7% higher for males), a higher prevalence in females than males already emerged in grade 4, where the between-gender difference (5.9%) was even higher than in grade 8 (4.1%) and 9 (5.5%). Strikingly, in grade 10, females exhibited a 20.0% higher myopia prevalence than males. Males were slightly, though non-significantly [all Holm-corrected *ps* > 0.05 (32)], older than females in all grades of S2 – thus, age differences between genders do not underly the higher myopia prevalence in females.

As myopia is often linked to academic achievement in the literature, we also examined myopia prevalence in the different schools – and thus school types – in the older sample (S2; see Table 3). Sorted from lowest to highest-level school leaving certificate that can be achieved at the respective schools, we found a 3.1% lower myopia prevalence in the general secondary school than in the intermediate secondary school, whose myopia prevalence was virtually the same as in the grammar school. Interestingly, the comprehensive school – offering all school leaving certificates – exhibited the highest myopia prevalence, which was even 3.2% higher than that in the grammar school. Thus, no clear picture emerged, as between-school differences – albeit following the order one might expect – were only marginal, and the comprehensive school exhibited the highest myopia prevalence. Please note the higher age of participants in the general secondary school (see Table 3). The same picture emerged for all grades individually.

**Table 3:** Myopia prevalence in S2 by school.

	Myopia				High myopia
Sample	Age *M* (SD)	*N*	% ≤ −0.75D	% ≤ −0.5D	% ≤ −6.0D
GSS	15.61 (1.14)	218	16.1	21.6	1.4
ISS	14.93 (1.03)	308	19.2	29.9	0.3
CS	14.71 (1.05)	287	22.6	34.1	0.0
GS	14.82 (1.06)	217	19.4	27.6	1.4

Age and N are presented for the sample included in the myopia prevalence calculation. For the high myopia prevalence calculation, 10 participants were excluded as described in data analysis. The age of participants included in the high myopia calculation was comparable to those included in the myopia calculation (see Table 1). GSS = general secondary school, ISS = intermediate secondary school, CS = comprehensive school, GS = grammar school.

**Figure 2:**
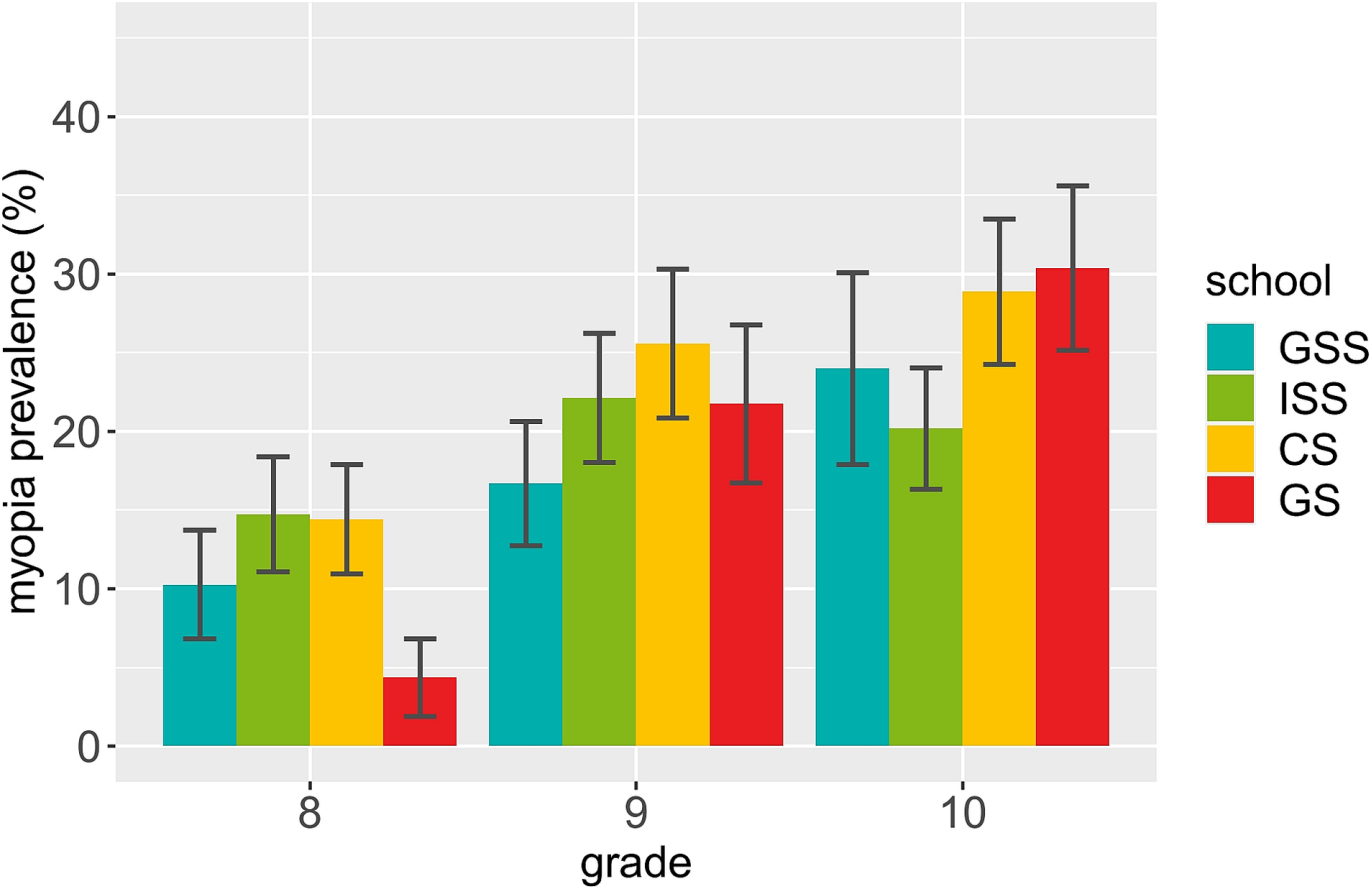
Myopia prevalence and standard error per school by grade in S2. GSS = general secondary school, ISS = intermediate secondary school, CS = comprehensive school, GS = grammar school.

Regarding individual grades per school type in the older sample (S2), there was again no clear picture, but an interesting pattern (see Figure 2): While myopia prevalence was higher for grade 10 than 8 in all schools, the magnitude of this difference varied, with the grammar school exhibiting the largest prevalence difference by far as well as the lowest myopia prevalence of all schools in grade 8. This is somewhat consistent with the frequently reported link between academic achievement and higher myopia prevalence. These findings should, however, be considered with caution, and need further investigation in a sample including more than one school per school type – as, e.g., social index levels varied between schools and may pose potential confounders.

### 3.2 Refractive status associations

**Figure 3:**
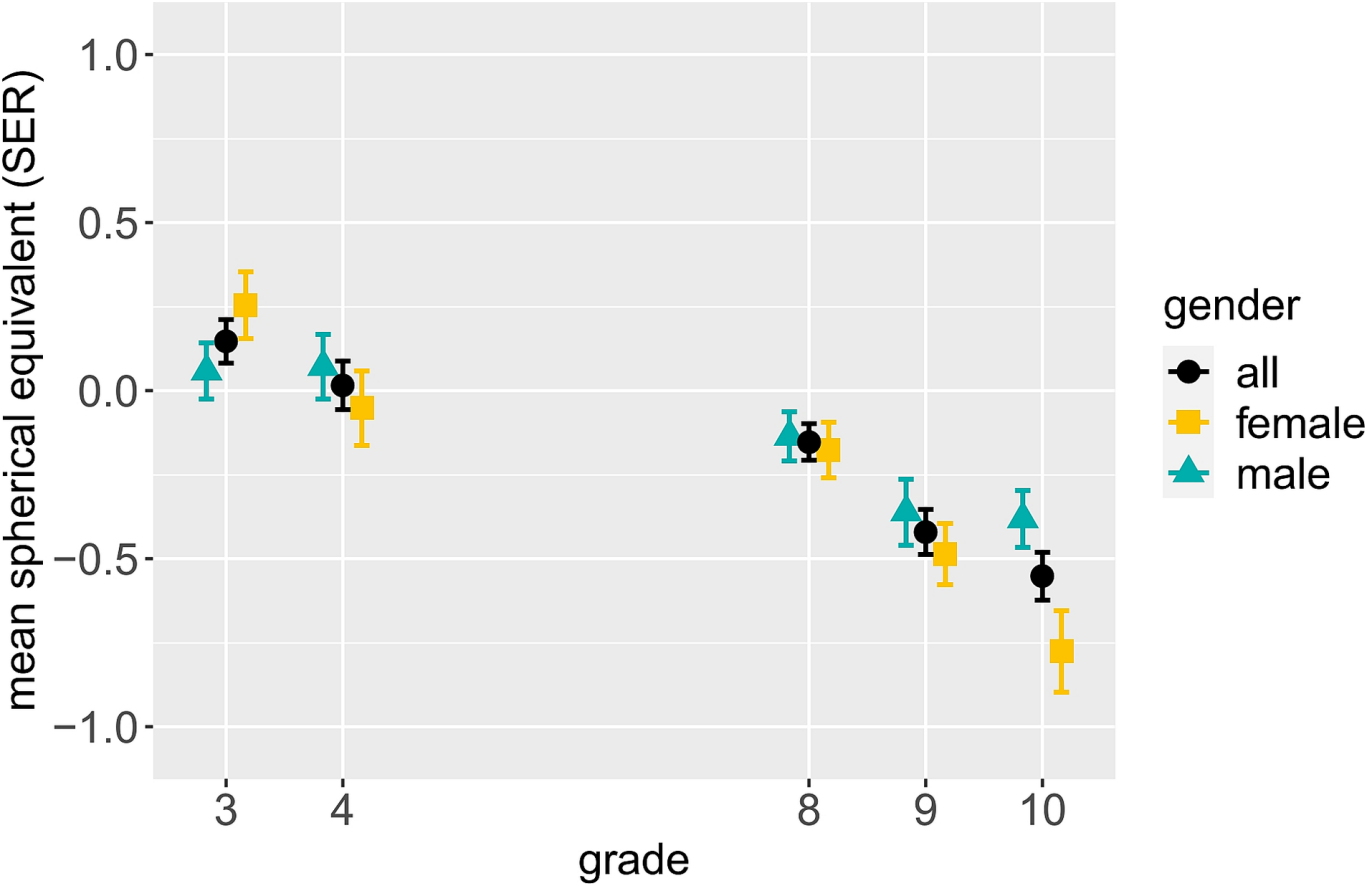
Mean SER and standard error per gender by grade. The data for all genders include eight more participants than the data of males and females combined due to four non-binary participants and four participants with unknown gender.

To consider a continuous variable sensitive to a person’s myogenic development prior to becoming myopic, we conducted further analyses with the SER, which was significantly more myopic in the older (S2, *N* = 1,029) than the younger (S1, *N* = 486) sample (*p* < 0.001; S1: *M* = 0.08D, SD = 1.06D; S2: *M* = −0.37D, SD = 1.20D). Figure 3 displays the mean SER per gender by grade. In concordance with the prevalence data presented in Figures 1, a more myopic SER is visible in higher than lower grades. Furthermore, a substantial mean SER difference of almost 0.4D between females and males is apparent in grade 10, with the females’ mean SER being more myopic.

Using multiple regression analysis, we found no associations with SER in the younger sample (S1, *N* = 483): The model with the predictors grade and gender did not reach statistical significance (*R^2^* = 0.004, *p* = 0.371). In the “all possible subsets” approach, the best-fitting model did not include any of the given predictors. The best-fitting model that included predictors and exhibited the lowest BIC and a comparatively high adjusted *R^2^* (see Methods for an explanation of the statistical terms) included only grade as predictor, but did not reach statistical significance (*R^2^* = 0.004, *p* = 0.175).

In the older sample (S2, *N* = 1,015), both grade and gender were significant predictors of SER (see Table 4; model A), with the respective model being overall significant (*R^2^* = 0.025, *p* < 0.001). This model was also identified as most promising via the “all possible subsets” approach. It exhibited the second-lowest BIC, which was only minimally higher than the lowest one, and the highest adjusted *R^2^* within a reasonable BIC range. The first model with a higher adjusted *R^2^* than model A included grade, gender and grade × gender as predictors (model B). It was thus also fitted, despite a substantial BIC difference to model A. Model B explained variance in SER (*R^2^* = 0.029, *p* < 0.001), but while grade was a significant predictor (grade 9: *p* = 0.023; grade 10: *p* < 0.001), gender and both grade × gender terms were not significant. Lastly, an F-test for nested models showed that model B did not fit the data better than model A (*p* = 0.139).

**Table 4:** Coefficient estimates of the multiple linear regression model A for S2.

Coefficient	*B*	95% CI	SE	*t*	*p*
Intercept	−0.26	[−0.41, −0.11]	0.08	−3.36	< 0.001
Grade 9	−0.26	[−0.43, −0.08]	0.09	−2.83	0.005
Grade 10	−0.40	[−0.58, −0.22]	0.09	−4.32	<0.001
Gender	0.18	[0.04, 0.33]	0.08	2.44	0.015

CI = confidence interval, SE = standard error.

Subsequent data inspection revealed a more myopic SER in females than males in S2 (females: *M* = −0.48D, SD = 1.22D, males: *M* = −0.29D, SD = 1.17D). Regarding grade, post-hoc Welch two sample t-tests [corrected with Holm’s method for multiple comparisons (32)] showed that the SER of grade 9 (*M* = −0.42D, SD = 1.25D) and grade 10 (*M* = −0.55D, SD = 1.29D) participants was significantly more myopic than that of grade 8 participants (*M* = −0.15D, SD = 1.02D; grade 8 vs. 9: *p* = 0.004; grade 8 vs. 10: *p* < 0.001). There was no significant difference between the SER of grade 9 and 10 participants (*p* = 0.183). Detailed statistical parameters for the calculations reported in this section are presented in Supplementary materials S5.

These results are in line with those on myopia prevalence rates (*cf.* Figure 1) and indicative of gender differences in myopic development being more present in older than younger children. Furthermore, grade significantly predicting SER in the older sample (S2), but not the younger one (S1), is consistent with the acceleration of myogenic development in teenage years. Yet, it should be noted that S1 encompassed two grades, and S2 three, thus impeding between-sample comparisons with regard to results on grade. Detailed results of the “all possible subsets” analyses can be found in Supplementary Tables S24, S25.

### 3.3 Uncorrected myopia

**Figure 4:**
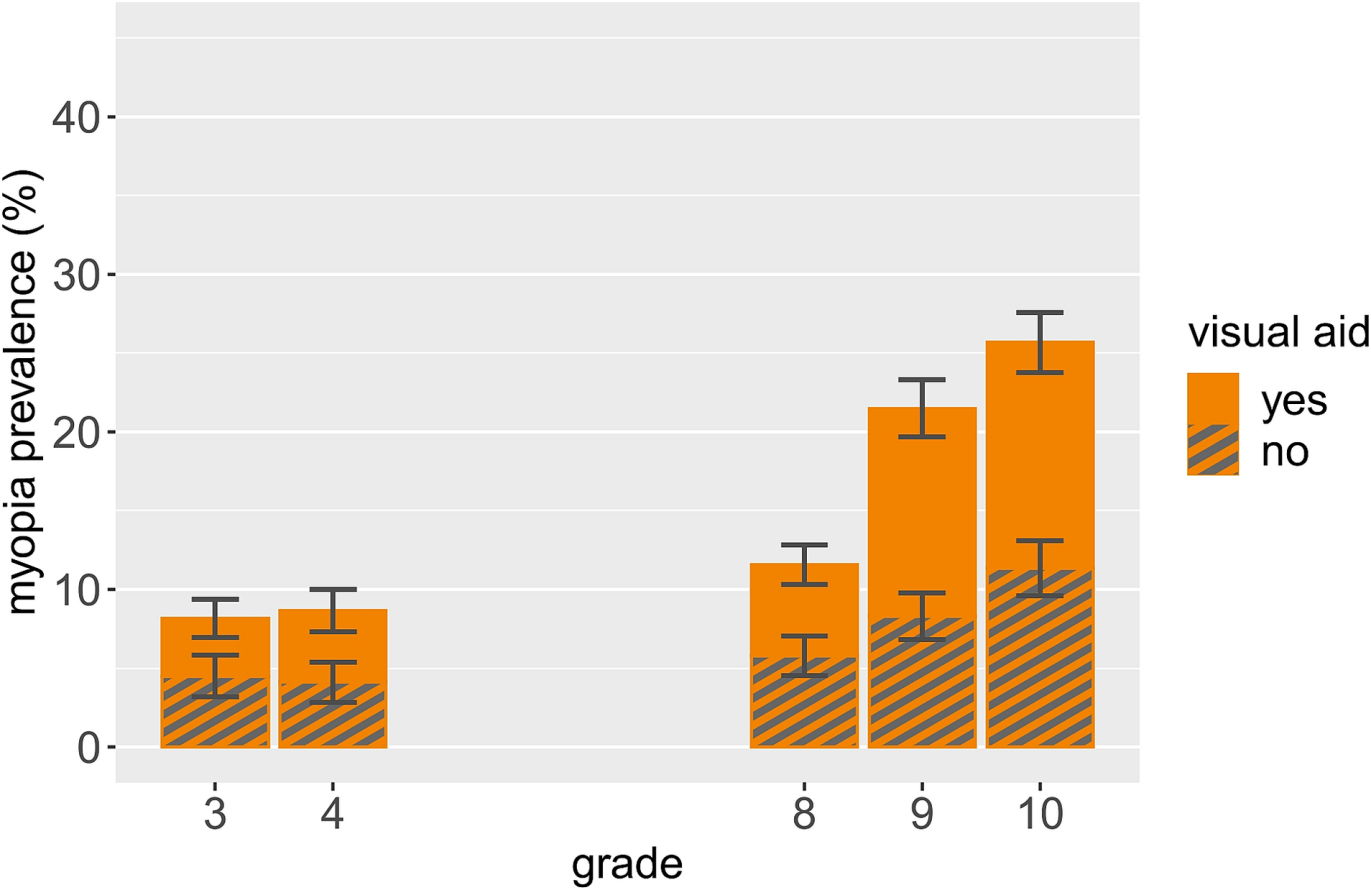
Corrected and uncorrected myopia prevalence and standard error by grade relative to the overall sample.

Shockingly, 51.2% of the 41 myopic participants in our younger sample (S1), and 43.3% of the 201 myopic participants in our older sample (S2) reported no visual aid. For the more conservative SER ≤ -1D myopia cut-off, these numbers were still as high as 48.7% (S1) and 32.7% (S2). These values are also presented in Table 5, together with the prevalence rates of uncorrected myopia per grade, showing that while there is some variation between grades, said prevalence does not systematically change with increasing grade, but is relatively stable across grades. Furthermore, Figure 4 shows the prevalence of corrected and uncorrected myopia per grade relative to the overall sample. It is readily apparent that based on the overall sample, the prevalence of uncorrected myopia is increased in higher compared to lower grade level, cumulating in more than 10% of all grade 10 participants having uncorrected myopia.

**Table 5:** Prevalence of uncorrected myopia in S1 and S2 Overall and by grade.

Sample	Myopia cut-off SER ≤ −0.75D		Myopia cut-off SER ≤ -1D	
	*N*	% Uncorrected	*N*	% Uncorrected
S1	41	51.2	39	48.7
Grade 3	20	55.0	18	50.0
Grade 4	21	47.6	21	47.6
S2	201	43.3	159	32.7
Grade 8	40	50.0	29	37.9
Grade 9	75	38.7	61	29.5
Grade 10	86	44.2	69	33.3

N indicates the number of myopic participants per the respective cut-off. The prevalence indicates the percentage of myopic participants without visual aid based on all myopic participants.

With regard to gender, the prevalence of uncorrected myopia was 55.0% for males and 47.6% for females in the younger sample (S1), and 44.7% for males and 42.5% for females in the older sample (S2). With the SER ≤ -1D myopia cut-off, these numbers were 52.6% (30.8%) for males and 45.0% (34.1%) for females in S1 (S2). Thus, the pattern we found for myopia prevalence regarding grade and gender does not emerge for the prevalence of uncorrected myopia (based on all myopic participants).

Lastly, of the myopic participants in the younger sample (S1), 38.9% (7 of 18) in the three schools with the lowest social index levels – i.e., lower social burden – and 60.9% (14 of 23) in the three schools with the highest social index levels – i.e., higher social burden – were uncorrected. This was only assessed in S1, as social index level is confounded with type of school in S2. Since data on (un)corrected myopia in S1 are based on 41 myopic participants only, this finding should be considered with caution and warrants replication.

## 4 Discussion

We investigated myopia prevalence, potential associations with SER, and prevalence of uncorrected myopia in school students in Germany. Myopia prevalence was 8.4% for grade 3–4 primary school students (S1) and 19.5% for grade 8–10 secondary school students (S2), with a substantial difference between grade 8 (11.6%) and grades 9 (21.5%) and 10 (25.7%). Apart from one exception, myopia prevalence was higher in higher versus lower grades for all schools in S2 – but the magnitude of this difference varied between schools, and thus types of schools. The grammar school exhibited the largest prevalence difference between grades 8 and 10. In S2, we also found a 10% higher myopia prevalence in females than males, with a higher magnitude of the gender difference in higher than lower grades. Neither age, gender, grade nor their interactions predicted SER in multiple linear regression analyses for S1. For S2, the model with grade and gender performed best, with more myopic SER in grades 9 and 10 than in grade 8 as well as in females than males. In S1, 51.2% of myopic participants were uncorrected, as were 43.3% in S2. More than 10% of the total grade 10 sample exhibited uncorrected myopia. Schools with a lower social burden exhibited a lower percentage of uncorrected versus corrected myopic participants than schools with a higher social burden in S1.

Table 6 presents myopia prevalence rates in children and adolescents from this study as well as other recent German and European investigations. The other German investigations (14, 42) report prevalence rates largely similar to the present ones – although one study yielded a 22% myopia prevalence in grade 5–7 grammar school students (42). This is interesting as participants in the respective study (42) were younger than those in our S2, and we measured an extremely low myopia prevalence in grade 8 grammar school students (4.3%, see Figure 2). Methodological differences might have played a role: In contrast to our opt-out procedure, active parental consent was conditional for participation in the respective study (42). Furthermore, while in said study, refraction was also measured objectively without cycloplegia, the myopia cut-off was more liberal than ours, and additional subjective refraction was often performed (42). Despite these differences that might benefit from further investigation, current data from Germany are generally consistent with our results.

**Table 6:** Myopia prevalence rates reported in this and other European studies.

Country	Cycloplegia	Myopia cut-off (SER)	Age (years)	Sample size	Myopia (%)
Germany (present study)	No	≤ −0.75D	9.30 (0.78)	488	8.4
		≤ −0.5D			11.3
		≤ −0.75D	14.99 (1.12)	1,030	19.5
		≤ −0.5D			28.8
Germany (42)	No	≤ −0.5D	11.2 (1.1)	274	22.3
Germany (14)	No	< −0.75D	8	342	3.9
			9	366	6.9
			10	349	11.1
			13	334	21.7
			14	301	22.8
			15	279	23.6
			16	213	26.4
Austria (43)	No	< −0.5D	15- < 18	1,507,063	24.8 (males)
Bosnia and Herzegovina (44)	Yes	≤ −0.5D	8	88	7.9
			9	123	7.3
			10	119	14.3
			13	114	21.5
			14	113	23.4
			15	101	28.2
			16	103	29.6
Denmark (36)	Yes	≤ −0.5D	15.4 (0.7)	307	17.9
	No				33.6
France (11)	Yes	≤ −0.5D	0–9	1,489	19.6
			10–19	8,289	42.7
Ireland (13)	Yes	≤ −0.5D	6.7 (0.49)	728	3.7
			12.8 (0.48)	898	22.8
Netherlands (10)	Yes	≤ −0.5D	6	5,711	2.4
Norway (12)	Yes	≤ −0.5D	16	246	11.0
Poland (45)	Yes	≤ −0.5D	≥6- < 9	4,875	3.65 (boys) 3.35 (girls)
			≥9- < 13		5.71 (boys) 8.30 (girls)
			≥13- < 16		5.96 (boys) 10.37 (girls)
Spain (46)	No	< −0.5D	6.19 (0.78)	1,993	19.1

The studies are chosen due to them reporting data from Germany (14, 42) or their inclusion in one of the reviews discussed earlier (4, 9). Data for the age groups corresponding best to the present study’s samples (S1: ca. 8–10 years; S2: ca. 13–16 years) are reported. In (46), data from 2016 and 2017 is reported, of which only the latter is included here. Age is reported as mean (SD) if possible. Otherwise, the age range is reported. Note that the publications partly differ in methodological aspects not reported here. For example, different methods have been used to assess refractive status, and the eyes on which the myopia cut-off was applied to vary (e.g., left, right, both, either), which may account for some variation in prevalence rates.

Results from many of the other European investigations are also overall in agreement with ours (10, 13, 36, 43, 44), especially considering methodological differences and the usual myopia prevalence increase during school age. Likewise, the low prevalence of high myopia in our data is consistent with the few other publications investigating high myopia (11–13).

Some European studies, however, differ more strongly from ours, yielding both lower (12, 45) and higher myopia prevalence rates (11, 46). One reason could be methodological differences: The much higher prevalence in Alvarez-Peregrina et al. (46) may partly be accounted to the fact that while in both, theirs and our study, non-cycloplegic measurements were taken, Alvarez-Peregrina et al. (46) used a myopia cut-off of < −0.5D SER. But even with a ≤ −0.5D cut-off, myopia prevalence in our S1 is still considerably lower than in their sample, despite the latter being younger. The authors suggest a potential bias due to their campaign offering free glasses if needed (46). Matamoros et al. (11) also report much higher prevalence rates than our study or others from Europe. Their data stem from eye clinics dedicated to refractive errors, so there may again be participation bias (11). Despite methodological aspects potentially explaining some differences, myopia prevalence likely also varies between countries based on other factors, and more research is needed to uncover those. This is especially important in light of the Covid-19 pandemic, as a recent meta-analysis shows accelerated myopic progression during compared to before the pandemic (47).

Regarding gender, we found a higher myopia prevalence and more myopic refractive status in females than males in our older sample (S2). This corresponds to prior results: One study, e.g., found a similar SER for Polish boys and girls before the age of 9, but lower a SER and higher myopia prevalence in females than males after that, with the prevalence of myopia being nearly twice as high in females than males aged 13–16 years (45). In their review, Rudnicka et al. (18) conclude that in white (and East Asian) populations, gender differences in myopia prevalence emerge around the age of 9 and become more pronounced thereafter, up to an odds ratio of myopia of about 2 for female versus male 17-18-year-olds. We also observed a higher between-gender prevalence difference among the older (grade 10) than younger (grade 8) participants in our S2. Furthermore, when adding grade × gender interactions to our regression model for predicting SER in S2, the interaction term for grade 10 was close to significance (see Supplementary materials S5) – even though the model did not outperform the model without the interaction. Our data thus support the notion of more pronounced gender differences in myopia prevalence in older than younger adolescents. Overall, both behavioral and biological factors may contribute to the higher myopia prevalence rates in female than male adolescents: For example, increased emphasis on educational activities and near work in girls, compared to boys, has repeatedly been suggested as a behavioral factor (18, 45, 48). A somewhat contrasting example substantiates the influence of such behavioral factors, as boys attending Orthodox Jewish schools with intensive education starting at an early age have been found to exhibit higher myopia prevalence rates compared to their peers, including girls (3, 48). On the other hand, myopia has also been associated with growth spurts and puberty (49–51), and respective timing differences in development between girls and boys may also partly explain the higher myopia prevalence rates in female compared to male adolescents (48).

Further interesting observations regarding grade were made. Firstly, we found a markedly higher myopia prevalence difference between grades 8 and 9 than grades 9 and 10. Furthermore, the prevalence in grade 8 is only 3% higher than in grade 4, but 9.9% lower than in grade 9. While grades 8 and 9 lie at the upper end or even beyond the 8–14 years of age during which school myopia typically appears (15), this result indicates that a large portion of myopia onset may happen between grades 8 and 9 in a German-like school system (with school entry at age 6 or 7). Although this should ideally be tested longitudinally, the present study did include a high number of participants. Considering the economic and personal burdens associated with uncorrected and/or high myopia (8, 20), this may well have public health implications. It may for example be reasonable to implement routine myopia assessments or health education on the importance of refractive correction in grade 9. During data collection, many uncorrected myopic participants in our older sample (S2) confirmed not seeing well, but expressed unwillingness to wear a visual aid due to concerns about their appearance and their peer group’s reaction – while at the same time having virtually no knowledge on myopia (implications). Thus, it may be helpful to target peer groups with interventions tailored to adolescents’ specific needs.

Secondly, the difference in myopia prevalence between grades 8 and 10 is especially pronounced for the grammar school, which offers the highest school leaving certificate, and also exhibits the lowest prevalence in grade 8 compared to the other secondary schools. This finding cannot be attributed to younger age of grammar school students: Students’ age was similar between all secondary schools but the general secondary school. If there is, in fact, a lower myopia prevalence in grade 8 grammar school students compared to other students, uncovering the underlying factors would be interesting. Yet, a much higher myopia prevalence for even younger grammar school students in Germany has also been reported (42), so this finding is far from conclusive. Meanwhile, we generally found little prevalence difference between secondary schools, which is maybe expected, since our participants from different schools but within the same grades had generally visited school for the same amount of years. The commonly reported link between academic achievement and myopia may be more pronounced later in life, when time spent on schooling differs more between people pursuing higher education versus not. Yet, students in Chinese elementary key (i.e., university-oriented) schools exhibited a higher myopia prevalence than students in less academically oriented, non-key schools – with a similar prevalence in grade 1, but a faster acceleration in key than non-key schools thereafter (52) – showing that there can be potentially education-related myopia prevalence differences even between students of similar grades. This result somewhat mirrors our finding of the highest between-grade prevalence difference in grammar school compared to other schools. Importantly, only one secondary school per school type was included in the investigation, and the calculations’ standard errors were large (see Figure 2). Said findings should thus be considered preliminary indications, as they may also be a result of other between-school differences. Still, they indicate potential interactions between school type, grade and myopia, which should be investigated further in samples better suited for respective analyses.

Analyses of SER generally confirmed the patterns observed with regard to myopia prevalence. The finding of neither age, grade or gender predicting SER in our younger sample (S1) can likely be attributed to the fact that both school myopia and gender differences in myopia prevalence start emerging only after the age of about eight or 9 years in samples such as ours (15, 17, 18). It should also be noted that S1 encompassed one less grade – and accordingly fewer variance in the variables grade and age – than our older sample (S2), so the SER analyses are not directly comparable between samples.

A striking finding of this investigation is the high prevalence of myopic participants that were uncorrected – specifically, 51.2% of myopic participants in our younger sample (S1) and 43.3% of myopic participants in our older sample (S2) did not have or report having a visual aid. Even when only considering participants with SER ≤ -1D, rates of uncorrected myopia were still 48.7% (S1) and 32.7% (S2). High rates of uncorrected and/or undetected myopia have been reported elsewhere: In a sample of Hong Kong primary school students (grade 1–6), only 23.6% of parents knew about their child’s refractive error (35). In Eastern China, 34.5% of myopic participants ranging from kindergarten to high school did not wear glasses (38), and in 6–8- and 11-13-year-olds in Canada, the rate of myopic participants that were uncorrected was also 34.5% (53). In 7-16-year-olds in Bosnia and Herzegovina, 54.5% of the study population required, but did not have refractive correction (44). These troubling results underline the necessity of early and repeated myopia screenings, which may contribute to reducing the high amount of visual impairments attributable to uncorrected refractive error (20). While there are mandatory vision screenings for school-aged children in some countries – for example, 41 US states require a vision screening for school-aged children, with between-state variation regarding frequency and timing (54, 55) – this is not the case everywhere. In Germany, a vision screening is conducted in the mandatory school entry examination prior grade 1 (around age 6). Only a few federal states have mandatory health examinations including a vision screening at some point during school age after that. The next nationwide mandatory vision screening is conducted when attempting to obtain a driver’s license (usually around the ages 16–18; though it is of course not mandatory to obtain a driver’s license). Considering the high prevalence of uncorrected myopia reported for school-aged children, the frequent lack of mandatory vision or refractive screenings is of concern – especially since school myopia onset usually lies between the ages of 8 and 14 years (15), and the absence of routine eye checks has been identified as a risk factor of myopia development in school students (35). Therefore, refractive screening at school age would be highly advisable. Given the large prevalence difference between grade 8 and 9 in the present study, these screenings should not discontinue before grade 9 – but should also be performed in (later) adolescence. As stated before, education about refractive errors, their potential consequences and correction could also be helpful to raise societal awareness, and might with appropriate peer group interventions increase adolescents’ acceptance of visual aids.

Lastly, the rate of myopic participants without correction was 22% lower in primary schools with low than in those with high social burden. While these results are preliminary and the low sample size should be considered when interpreting them, it may be worthwhile to test potential associations between uncorrected myopia and social burden in a larger sample. If such a result can in fact be replicated, this may be another potential aspect one could incorporate in the planning of refractive screenings or health education with regard to refractive errors.

### 4.1 Strengths and limitations

A strength of this study is sample representativeness, achieved through contacting schools in the area in a random order and an inclusion of the different types of schools. Among others, the variance in social index levels confirms some variability between participating schools. Representativeness was further increased by using non-invasive autorefraction and immediate data anonymization – as therefore, the need for informed parental consent was waived by the ethics committee. Instead, an opt-out procedure was used in that participants or their caregivers could refuse participation. Had we used more invasive methods, for example cycloplegia, active parental consent would have been necessary. This would most likely have entailed a significantly lower participation rate as well as a participation bias, with a potential underrepresentation of specific social groups.

On the other hand, the use of non-cycloplegic refraction measurements also poses a limitation of our study, since they have been shown to measure a more myopic SER and thus overestimate myopia as compared to cycloplegic refraction measurements (9, 18). However, good measurement accuracy has been reported for non-cycloplegic measurements with Plusoptix devices before, especially in non-hyperopic individuals (56–59). For example, in children as young as 7.63 ± 3.41 years, the mean SER of non-cycloplegic Plusoptix A12 measurements was only 0.43D more myopic than that of cycloplegic refraction measurements. Thereby, the mean difference between Plusoptix and cycloplegic measurements was −0.048D for the myopic and 0.37D for the hyperopic spherical component (56). A recent systematic review and meta-analysis confirms a generally reasonable agreement between non-cycloplegic Plusoptix measurements and cycloplegic measurements (60). Taken together with the fact that the difference between cycloplegic and non-cycloplegic measurements is both higher in younger than older participants (18) and especially strong in more hyperopic individuals (61–63) as well as our use of a ≤ −0.75D SER myopia definition, we assume that the use of non-cycloplegic measurements did not overly distort our prevalence estimates in our older sample (S2). In younger participants, deviations of non-cycloplegic from cycloplegic measurements are generally larger. Yet, investigations showing a good measurement accuracy of non-cycloplegic Plusoptix measurements and reasonable agreement with cycloplegic refraction were often conducted with young participants (56, 60). Also, the participants classified as myopic in our younger sample (S1) had a mean SER of −2.52D ± 1.41D, with > -1D in only two participants. We thus suspect that the overestimation of myopia prevalence in our S1 was not as grave either.

Another limitation is the inclusion of only one secondary school per school type, which gravely limits the informative value of results regarding different types of schools, as the respective schools also differed in characteristics like their social index levels. Yet, we did not want to omit the findings regarding the different schools in S2, but we explicitly emphasize that they may be confounded and should be interpreted as tentative, non-conclusive indications.

Lastly, the detected measurement differences between the two autorefractometer models are of course very unfortunate. We have taken steps to correct for these by linearly transforming the data from the deviating device and the additional analyses in the Supplementary materials confirm that, both compared to the data before the linear transformation and to the data when the deviating device is excluded, the results do not show much change. Thus, while this circumstance is undesirable and should be avoided in the future, there is likely no major impact from the between-model deviation.

### 4.2 Conclusion

The 8.4% prevalence we observed for 3rd- and 4th-graders (S1) as well as the 19.5% prevalence we observed for 8th-, 9th- and 10th-graders (S2) in Germany are generally in line with other European investigations. Furthermore, the higher prevalence and more myopic SER in S2 than S1 as well as in higher versus lower grades within S2 was as expected. With regard to specific grades, our results show that grades 8 and 9 – i.e., around the ages 13–15 – seem to be an important time with regard to myopia onset. In accordance with other investigations, our data also demonstrate a higher myopia prevalence and a more myopic refractive status in females than in males in the older sample, accelerating with increasing grade. Lastly, we found a strikingly high proportion of uncorrected (versus corrected) myopia in both samples, and more than 10% of the complete grade 10 sample had uncorrected myopia. These drastic results warrant further consideration and call for interventive measures. Generally, our findings entail important implications for public health – specifically, they underline the necessity of mandatory refractive screenings and health education on the implications of myopia for school-aged children and adolescents.

## Data Availability

The raw data supporting the conclusions of this article will be made available by the authors, without undue reservation.
