# Supplementary material for: Myopia prevalence, refractive status and uncorrected myopia among primary and secondary school students in Germany
Source: Front Med (Lausanne). 2024 Dec 12;11:1483069. doi: 10.3389/fmed.2024.1483069 (PMC11669525; doi:10.3389/fmed.2024.1483069)
Supplement: Supplementary file 1 [file Data_Sheet_1.docx]

Supplementary Material to:

Myopia Prevalence, Refractive Status and Uncorrected Myopia Among Primary and Secondary School Students in Germany

# Device A09 Data Transformation

*In the following, we present results on the data preparation and A09 device data transformation based on the comparison study.*

Supplementary Table 1

*Comparison Study SER Measurements*

| **device** | ***M*** | ***SD*** |
| --- | --- | --- |
| right eye | | |
| A09 | -0.53D | 1.41D |
| A12R-1 | -0.80D | 1.33D |
| A12R-2 | -0.82D | 1.24D |
| left eye | | |
| A09 | -0.50D | 1.47D |
| A12R-1 | -0.71D | 1.37D |
| A12R-2 | -0.75D | 1.25D |

*Note.* Data from 58 participants.

Supplementary Table 2

*T-test Results Comparing Devices’ SER Measurements in Comparison Study*

| **devices** | ***t*** | ***df*** | **95% CI** | ***p*** |
| --- | --- | --- | --- | --- |
| right eye | | | | |
| A09 vs. A12R-1 | 8.20 | 57 | [0.20, 0.34] | < .001 |
| A09 vs. A12R-2 | 6.78 | 57 | [0.20, 0.38] | < .001 |
| A12R-1 vs. A12R-2 | 0.84 | 57 | [-0.03, 0.07] | .404 |
| left eye | | | | |
| A09 vs. A12R-1 | 7.46 | 57 | [0.16, 0.27] | < .001 |
| A09 vs. A12R-2 | 5.47 | 57 | [0.16, 0.35] | < .001 |
| A12R-1 vs. A12R-2 | 1.50 | 57 | [-0.01, 0.10] | .278 |

*Note.* CI = confidence interval. Holm-corrected p-values are reported.

**
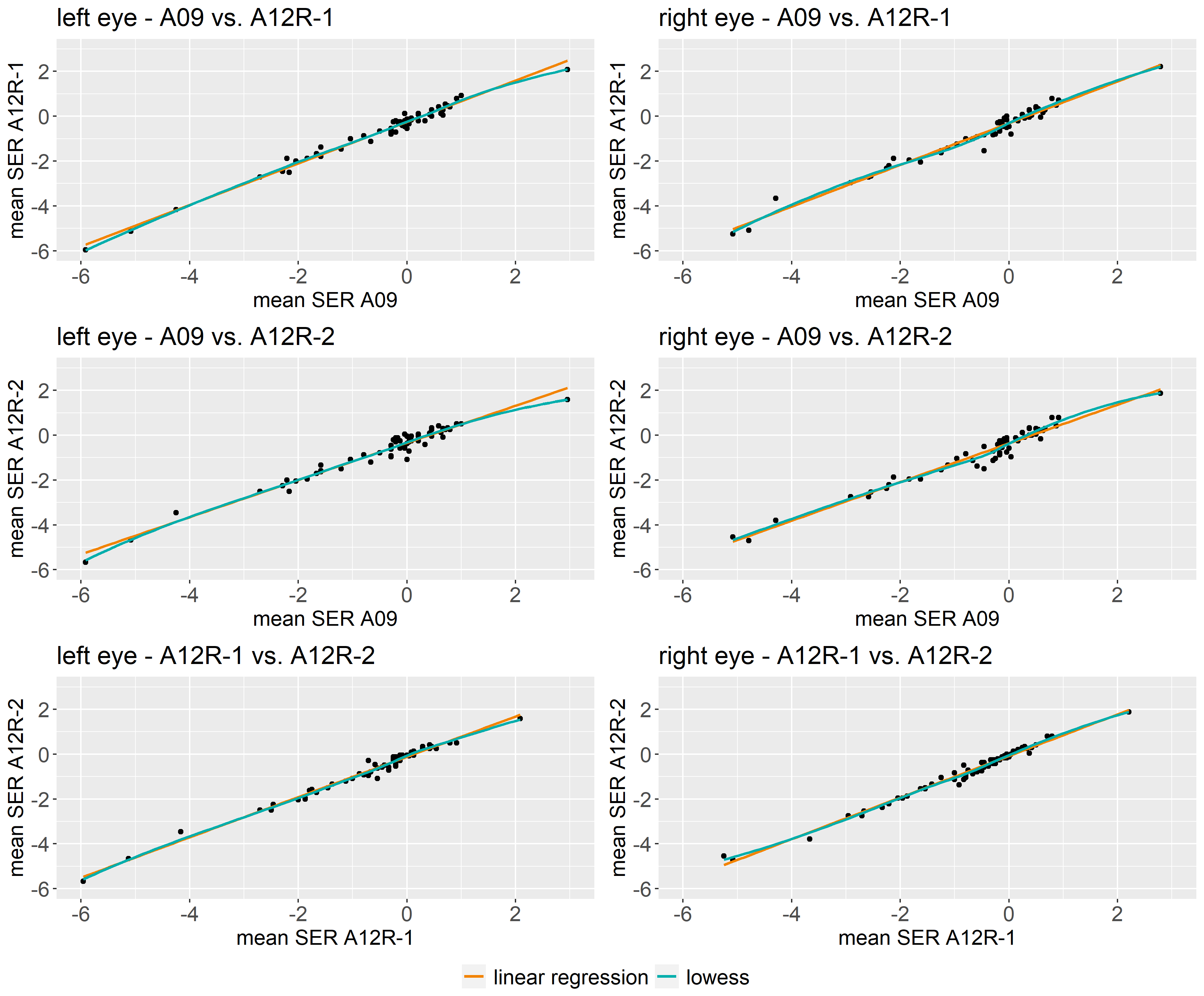
**

Supplementary Figure 1. Linear regression and LOWESS lines for the mean SER data for each device pair in comparison study.

Supplementary Table 3

*GAMs With GCV to Predict Either A12R Mean SER From A09 Mean SER*

| **A12R-1** | | | | | |
| --- | --- | --- | --- | --- | --- |
| **right eye** | | | | | |
| component | term | estimate | SE | *t* | *p* |
| parametric coefficients | intercept | -0.22 | 0.09 | -2.36 | .022 |
|  | A09 mean SER | 1.10 | 0.16 | 6.73 | < .001 |
| component | term | *edf* | reference *df* | *F* | *p* (adjusted) |
| smooth term | s(A09 mean SER) | 4.00 | 8 | 1.03 | .197 |
| **left eye** | | | | | |
| component | term | estimate | SE | *t* | *p* |
| parametric coefficients | intercept | -0.25 | 0.03 | -7.96 | < .001 |
|  | A09 mean SER | 0.92 | 0.04 | 21.12 | < .001 |
| component | term | *edf* | reference *df* | *F* | *p* (adjusted) |
| smooth term | s(A09 mean SER) | 1.17 | 8 | 0.58 | .086 |
| **A12R-2** | | | | | |
| **right eye** | | | | | |
| component | term | estimate | SE | *t* | *p* |
| parametric coefficients | intercept | -0.40 | 0.08 | -5.13 | < .001 |
|  | A09 mean SER | 0.79 | 0.13 | 6.00 | < .001 |
| component | term | *edf* | reference *df* | *F* | *p* (adjusted) |
| smooth term | s(A09 mean SER) | 2.81 | 8 | 0.60 | .197 |
| **left eye** | | | | | |
| component | Term | estimate | SE | *t* | *p* |
| parametric coefficients | intercept | -0.34 | 0.06 | -5.27 | < .001 |
|  | A09 mean SER | 0.82 | 0.11 | 7.22 | < .001 |
| component | term | *edf* | reference *df* | *F* | *p* (adjusted) |
| smooth term | s(A09 mean SER) | 2.75 | 8 | 0.80 | .197 |

*Note.* GAM = generalized additive model. GCV = generalized cross-validation. SE = standard error. The models were fitted as described in the Methods section of the manuscript. P-values marked with “adjusted” are Holm-corrected due to multiple testing of four smooth terms.

Supplementary Table 4

*GAMs With REML Criterion to Predict Either A12R Mean SER From A09 Mean SER*

| **A12R-1** | | | | | |
| --- | --- | --- | --- | --- | --- |
| **right eye** | | | | | |
| component | term | estimate | SE | *t* | *p* |
| parametric coefficients | intercept | -0.31 | 0.03 | -9.42 | < .001 |
|  | A09 mean SER | 0.93 | 0.02 | 42.70 | < .001 |
| component | term | *edf* | reference *df* | *F* | *p* (adjusted) |
| smooth term | s(A09 mean SER) | 0.00 | 8 | 0.00 | .599 |
| **left eye** | | | | | |
| component | term | estimate | SE | *t* | *p* |
| parametric coefficients | intercept | -0.26 | 0.04 | -7.55 | < .001 |
|  | A09 mean SER | 0.91 | 0.05 | 18.66 | < .001 |
| component | term | *edf* | reference *df* | *F* | *p* (adjusted) |
| smooth term | s(A09 mean SER) | 1.39 | 8 | 0.65 | .093 |
| **A12R-2** | | | | | |
| **right eye** | | | | | |
| component | term | estimate | SE | *t* | *p* |
| parametric coefficients | intercept | -0.36 | 0.04 | -9.34 | < .001 |
|  | A09 mean SER | 0.86 | 0.03 | 25.41 | < .001 |
| component | term | *edf* | reference *df* | *F* | *p* (adjusted) |
| smooth term | s(A09 mean SER) | 0.26 | 8 | 0.04 | .586 |
| **left eye** | | | | | |
| component | term | estimate | SE | *t* | *P* |
| parametric coefficients | intercept | -0.35 | 0.05 | -7.77 | < .001 |
|  | A09 mean SER | 0.81 | 0.06 | 12.84 | < .001 |
| component | term | *edf* | reference *df* | *F* | *p* (adjusted) |
| smooth term | s(A09 mean SER) | 1.30 | 8 | 0.42 | .208 |

*Note.* GAM = generalized additive model. GCV = generalized cross-validation. SE = standard error. The models were fitted as described in the Methods section of the manuscript. P-values marked with “adjusted” are Holm-corrected due to multiple testing of four smooth terms.

Supplementary Table 5

*Linear Regressions to Predict A12R-1 and A12R-2 Mean SER From A09 Mean SER*

| **coefficient** | ***B*** | **95% CI** | **SE** | ***t*** | ***p*** |
| --- | --- | --- | --- | --- | --- |
| right eye | | | | | |
| intercept | -0.3346365 | [-0.40, -0.27] | 0.03 | -10.12 | < .001 |
| A09 mean SER | 0.8961972 | [0.85, 0.94] | 0.02 | 40.52 | < .001 |
| left eye | | | | | |
| intercept | -0.2957028 | [-0.35, -0.24] | 0.03 | -10.38 | < .001 |
| A09 mean SER | 0.8757312 | [0.84, 0.91] | 0.02 | 47.49 | < .001 |

*Note.* The mean SER of the A12R devices was averaged. For reasons of reproducibility, the linear transformation is reported as accurately as possible with seven decimal places of the point estimate. The formula *-0.3346365 + 0.8961972*A09 mean SER* was used for linear transformation of the A09 mean SER data for the right eye, and the formula *-0.2957028 + 0.8757312*A09 mean SER* for the left eye.

# Recalculated Results

*In the following, we present the results recalculated (1) for the complete data without linear transformation of the A09 device data (Supplementary Tables 6, 8, 10, 12, 14, 16, 18, 20 and 22; Supplementary Information S1 and S3) and (2) for the A12R devices’ data only (Supplementary Tables 7, 9, 11, 13, 15, 17, 19, 21 and 23; Supplementary Information S2 and S4).*

Supplementary Table 6

Myopia and High Myopia Prevalence in S1 and S2 Overall and by Grade for the Complete Data Without Linear Transformation of the A09 Device Data

|  | **myopia** | | | | **high myopia** |
| --- | --- | --- | --- | --- | --- |
| **sample** | **age *M*(*SD*)** | ***N*** | **% ≤ -0.75D** | **% ≤ -0.5D** | **% ≤ -6.0D** |
| S1 | 9.30 (0.78) | 488 | 8.4 | 10.7 | 0.4 |
| grade 3 | 8.85 (0.73) | 245 | 8.2 | 10.2 | 0.4 |
| grade 4 | 9.75 (0.53) | 243 | 8.6 | 11.1 | 0.4 |
| S2 | 14.99 (1.12) | 1030 | 18.4 | 24.8 | 0.8 |
| grade 8 | 13.98 (0.77) | 346 | 10.1 | 16.2 | 0.2 |
| grade 9 | 15.04 (0.80) | 349 | 21.0 | 25.5 | 0.6 |
| grade 10 | 15.97 (0.73) | 335 | 24.5 | 32.8 | 1.5 |

*Note.* Age and *N* are presented for the sample included in the myopia prevalence calculation. For the high myopia prevalence calculation, 2 (10) of these participants were excluded from S1 (S2) as described in the Data Analysis section of the manuscript. Thus, 486 participants (age: 9.29 ± 0.77 years) were included in the high myopia prevalence calculation for S1, as were 1020 participants (age: 14.98 ± 1.12 years) for S2. The corresponding data are presented in Table 1 in the manuscript. Here, the prevalence for the ≤ -0.5D myopia cut-off in grades 9 & 10 is a bit lower than in the manuscript’s data, but the general tendencies are similar.

Supplementary Table 7

Myopia and High Myopia Prevalence in S1 and S2 Overall and by Grade for the A12R Devices’ Data Only

|  | **myopia** | | | | **high myopia** |
| --- | --- | --- | --- | --- | --- |
| **sample** | **age *M*(*SD*)** | ***N*** | **% ≤ -0.75D** | **% ≤ -0.5D** | **% ≤ -6.0D** |
| S1 | 9.29 (0.75) | 342 | 8.2 | 11.4 | 0.3 |
| grade 3 | 8.85 (0.70) | 165 | 7.9 | 10.9 | 0.6 |
| grade 4 | 9.71 (0.51) | 177 | 8.5 | 11.9 | 0.0 |
| S2 | 15.03 (1.14) | 677 | 20.8 | 28.8 | 0.7 |
| grade 8 | 14.02 (0.82) | 225 | 11.6 | 18.7 | 0.0 |
| grade 9 | 15.08 (0.83) | 230 | 22.6 | 28.7 | 0.4 |
| grade 10 | 16.01 (0.75) | 222 | 28.4 | 39.2 | 1.8 |

*Note.* Age and *N* are presented for the sample included in the myopia prevalence calculation. For the high myopia prevalence calculation, 1 (7) of these participants were excluded from S1 (S2) as described in the Data Analysis section of the manuscript. Thus, 341 participants (age: 9.29 ± 0.74 years) were included in the high myopia prevalence calculation for S1, as were 670 participants (age: 15.03 ± 1.14 years) for S2. The corresponding data are presented in Table 1 in the manuscript.

Supplementary Table 8

*Myopia Prevalence in S1 and S2 by Gender for the Complete Data Without Linear Transformation of the A09 Device Data*

|  | **myopia** | | | | **high myopia** |
| --- | --- | --- | --- | --- | --- |
| **sample** | **age *M*(*SD*)** | ***N*** | **% ≤ -0.75D** | **% ≤ -0.5D** | **% ≤ -6.0D** |
| S1 |  |  |  |  |  |
| female | 9.27 (0.77) | 219 | 9.6 | 11.0 | 0.0 |
| male | 9.33 (0.79) | 266 | 7.5 | 10.5 | 0.8 |
| S2 |  |  |  |  |  |
| female | 14.90 (1.05) | 454 | 23.6 | 28.9 | 0.7 |
| male | 15.05 (1.17) | 571 | 14.0 | 21.2 | 0.9 |

*Note.* Four participants with no information on their gender and four non-binary participants were excluded from these calculations. Age and *N* are presented for the sample included in the myopia prevalence calculation. For the high myopia prevalence calculation, 2 (10) of these participants were excluded from S1 (S2) as described in the Data Analysis section of the manuscript. Thus, 483 participants (age: 9.30 ± 0.78 years) were included in the high myopia prevalence calculation for S1, as were 1015 participants (age: 14.98 ± 1.12 years) for S2. The corresponding data are presented in Table 2 in the manuscript. Here, the prevalence for the ≤ -0.5D myopia cut-off in S2 is a bit lower than in the manuscript’s data, but the general tendencies are similar.

Supplementary Table 9

*Myopia Prevalence in S1 and S2 by Gender for the A12R Devices’ Data Only*

|  | **myopia** | | | | **high myopia** |
| --- | --- | --- | --- | --- | --- |
| **sample** | **age *M*(*SD*)** | ***N*** | **% ≤ -0.75D** | **% ≤ -0.5D** | **% ≤ -6.0D** |
| S1 |  |  |  |  |  |
| female | 9.32 (0.77) | 154 | 10.4 | 12.3 | 0.0 |
| male | 9.28 (0.73) | 186 | 6.5 | 10.8 | 0.5 |
| S2 |  |  |  |  |  |
| female | 14.92 (1.10) | 294 | 26.9 | 33.0 | 0.7 |
| male | 15.12 (1.17) | 381 | 16.0 | 25.5 | 0.8 |

*Note.* Two participants with no information on their gender and two non-binary participants were excluded from these calculations. Age and *N* are presented for the sample included in the myopia prevalence calculation. For the high myopia prevalence calculation, 1 (7) of these participants were excluded from S1 (S2) as described in the Data Analysis section of the manuscript. Thus, 339 participants (age: 9.29 ± 0.74 years) were included in the high myopia prevalence calculation for S1, as were 668 participants (age: 15.03 ± 1.14 years) for S2. The corresponding data are presented in Table 2 in the manuscript.

Supplementary Table 10

*Myopia Prevalence and Standard Error per Gender by Grade for the Complete Data Without Linear Transformation of the A09 Device Data*

| **sample** | **myopia (% ≤ -0.75D) (SE)** | | |
| --- | --- | --- | --- |
|  | **female** | **male** | **all genders** |
| S1 |  |  |  |
| grade 3 | 7.3 (2.5) | 9.0 (2.5) | 8.2 (1.8) |
| grade 4 | 11.9 (3.1) | 6.1 (2.1) | 8.6 (1.8) |
| S2 |  |  |  |
| grade 8 | 11.9 (2.7) | 8.9 (2.0) | 10.1 (1.6) |
| grade 9 | 24.4 (3.3) | 17.8 (2.9) | 20.9 (2.2) |
| grade 10 | 34.3 (4.0) | 15.9 (2.7) | 24.5 (2.4) |

*Note.* SE = standard error. The data for all genders include eight more participants than the data of males and females combined due to four non-binary participants and four participants with unknown gender. The corresponding data are presented in Figure 1 in the manuscript.

Supplementary Table 11

*Myopia Prevalence and Standard Error per Gender by Grade for the A12R Devices’ Data Only*

| **sample** | **myopia (% ≤ -0.75D) (SE)** | | |
| --- | --- | --- | --- |
|  | **female** | **male** | **all genders** |
| S1 |  |  |  |
| grade 3 | 7.0 (3.1) | 8.6 (2.9) | 7.9 (2.1) |
| grade 4 | 13.3 (3.7) | 4.3 (2.1) | 8.5 (2.1) |
| S2 |  |  |  |
| grade 8 | 14.9 (3.7) | 9.2 (2.5) | 11.6 (2.1) |
| grade 9 | 28.8 (4.5) | 17.5 (3.4) | 22.6 (2.8) |
| grade 10 | 36.5 (4.9) | 21.6 (3.7) | 28.4 (3.0) |

*Note*. SE = standard error. The data for all genders include four more participants than the data of males and females combined due to two non-binary participants and two participants with unknown gender. The corresponding data are presented in Figure 1 in the manuscript. Here, the prevalence for female participants in grade 9 and for male participants in grade 10 is slightly higher in the manuscript’s data, and the latter is also higher than that of male participants in grade 9 (other than in the manuscript’s data) – however, the general tendencies are similar.

Supplementary Table 12

Myopia Prevalence in S2 by School for the Complete Data Without Linear Transformation of the A09 Device Data

|  | **myopia** | | | | **high myopia** |
| --- | --- | --- | --- | --- | --- |
| **sample** | **age *M*(*SD*)** | ***N*** | **% ≤ -0.75D** | **% ≤ -0.5D** | **% ≤ -6.0D** |
| GSS | 15.61 (1.14) | 218 | 15.1 | 19.3 | 1.4 |
| ISS | 14.93 (1.03) | 308 | 18.2 | 25.6 | 0.3 |
| CS | 14.71 (1.05) | 287 | 20.9 | 28.9 | 0.4 |
| GS | 14.82 (1.06) | 217 | 18.9 | 23.5 | 1.4 |

*Note.* Age and *N* are presented for the sample included in the myopia prevalence calculation. For the high myopia prevalence calculation, 10 participants were excluded as described in the Data Analysis section of the manuscript. The age of participants included in the high myopia calculation was comparable to those included in the myopia calculation (see Supplementary Table 1). GSS = general secondary school, ISS = intermediate secondary school, CS = comprehensive school, GS = grammar school. The corresponding data are presented in Table 3 in the manuscript. Here, the prevalence rates for the ≤ -0.5D cut-off are somewhat lower than in the manuscript’s data, but the general tendencies are similar.

Supplementary Table 13

Myopia Prevalence in S2 by School for the A12R Devices’ Data Only

|  | **myopia** | | | | **high myopia** |
| --- | --- | --- | --- | --- | --- |
| **sample** | **age *M*(*SD*)** | ***N*** | **% ≤ -0.75D** | **% ≤ -0.5D** | **% ≤ -6.0D** |
| GSS | 15.72 (1.15) | 146 | 17.1 | 21.9 | 1.4 |
| ISS | 14.95 (1.06) | 203 | 18.7 | 28.6 | 0.0 |
| CS | 14.69 (1.04) | 183 | 27.3 | 37.2 | 0.0 |
| GS | 14.88 (1.07) | 145 | 19.3 | 25.5 | 2.1 |

*Note.* Age and *N* are presented for the sample included in the myopia prevalence calculation. For the high myopia prevalence calculation, 7 participants were excluded as described in the Data Analysis section of the manuscript. The age of participants included in the high myopia calculation was comparable to those included in the myopia calculation (see Supplementary Table 2). GSS = general secondary school, ISS = intermediate secondary school, CS = comprehensive school, GS = grammar school. The corresponding data are presented in Table 3 in the manuscript. Here, the prevalence for the CS is slightly higher than in the manuscript’s data, but the general tendencies are similar.

Supplementary Table 14

*Myopia Prevalence and Standard Error in S2 per School by Grade for the Complete Data Without Linear Transformation of the A09 Device Data*

| **sample** | **myopia (% ≤ -0.75D) (SE)** | | | |
| --- | --- | --- | --- | --- |
|  | **GSS** | **ISS** | **CS** | **GS** |
| grade 8 | 10.3 (3.5) | 12.6 (3.4) | 11.5 (3.1) | 4.3 (2.5) |
| grade 9 | 15.6 (3.8) | 22.1 (4.1) | 24.4 (4.7) | 21.7 (5.0) |
| grade 10 | 22.0 (5.9) | 19.3 (3.8) | 27.8 (4.6) | 29.1 (5.1) |

*Note.* SE = standard error. GSS = general secondary school, ISS = intermediate secondary school, CS = comprehensive school, GS = grammar school. The corresponding data are presented in Figure 2 in the manuscript.

Supplementary Table 15

*Myopia Prevalence and Standard Error in S2 per School by Grade for the A12R Devices’ Data Only*

| sample | myopia (% ≤ -0.75D) (SE) | | | |
| --- | --- | --- | --- | --- |
|  | GSS | ISS | CS | GS |
| grade 8 | 9.8 (4.2) | 15.6 (4.6) | 15.2 (4.4) | 2.3 (2.3) |
| grade 9 | 16.9 (4.9) | 19.1 (4.8) | 33.3 (6.3) | 21.7 (6.1) |
| grade 10 | 27.8 (7.6) | 21.1 (4.9) | 35.0 (6.2) | 30.9 (6.3) |

*Note.* SE = standard error. GSS = general secondary school, ISS = intermediate secondary school, CS = comprehensive school, GS = grammar school. The corresponding data are presented in Figure 2 in the manuscript. Here, grade 9 ISS participants have a minimally lower prevalence than grade 9 GS participants, other than in the manuscript’s data. Also, the prevalence of CS participants in grades 9 & 10 is somewhat higher than in the manuscript’s data, and other than in the manuscript’s data, the prevalence of grade 10 CS participants is higher than that of grade 10 GS participants. Apart from that, the general tendencies are similar.

## Supplementary Information S1: SER Analyses for the Complete Data Without Linear Transformation of the A09 Device Data

SER was significantly more myopic in the older (S2, *N* = 1029) than the younger (S1, *N* = 486) sample for the complete data without linear transformation of the A09 device data (*t*(1513) = -6.88,
*p* < .001, *d* = 0.38, 95% CI [-0.59, -0.33]; S1: *M* = 0.19D, *SD* = 1.11D; S2: *M* = -0.26D, *SD* = 1.25D).

In the younger sample (S1, *N* = 483), the regression model with the predictors grade and gender did not reach statistical significance (*R^2^* = .006, *F*(2, 480) = 1.37, *p* = .255). In the “all possible subsets” approach, the best-fitting model did not include any of the given predictors. From the models including predictors, the best-fitting model included grade as only predictor, and it did also not reach statistical significance (*R^2^* = .005, *F*(1, 481) = 2.58, *p* = .109).

In the older sample (S2, *N* = 1015), both grade and gender were identified as significant predictors of SER (see Supplementary Table 18, model A), with the respective regression model being overall significant (*R^2^* = .024, *F*(3, 1011) = 8.46, *p* < .001). This model was also identified as the most promising one via the “all possible subsets” approach. The regression model including grade, gender and grade × gender as predictors (model B) exhibited the next-highest adjusted R^2^ and was thus also fitted, despite a substantial BIC difference to model A. Model B also explained variance in SER
(*R^2^* = .029, *F*(5, 1009) = 6.01, *p* < .001), but while grade (grade 9: *B* = -0.31, *p* = .029; grade 10:
*B* = -0.63, *p* < .001) and the gender × grade term for grade 10 (*B* = 0.38, *p* = .049) significantly predicted SER in this model (the latter other than in the manuscript’s data), gender and the gender × grade term for grade 9 did not reach significance (*B* = 0.05, *p* = .799). An F-test for nested models showed that model B did not fit the data better than model A (*F*(2, 1011) = 2.29, *p* = .102). SER was more myopic in females than males in S2 (females: *M* = -0.37D, *SD* = 1.26D, males: *M* = -0.18D,
*SD* = 1.22D). Post-hoc Holm-corrected Welch two sample t-tests showed that the SER of grade 9
(*M* = -0.32D, *SD* = 1.29D) and grade 10 (*M* = -0.45D, *SD* = 1.34D) participants was significantly more myopic than that of grade 8 participants (*M* = -0.03D, *SD* = 1.06D; grade 8 vs. 9:
*t*(662.52) = 3.16, *p* = .003, *d* = 0.24, 95% CI [0.11, 0.46]; grade 8 vs. 10: *t*(617.75) = 4.41, *p* < .001,
*d* = 0.34, 95% CI [0.23, 0.60]). There was no significant difference between the SER of grade 9 and 10 participants (*t*(662.38) = 1.26, *p* = .209, *d* = 0.10, 95% CI [-0.07, 0.33]).

## Supplementary Information S2: SER Analyses for the A12R Devices’ Data Only

SER was significantly more myopic in the older (S2, *N* = 677) than the younger (S1, *N* = 341) sample for the A12R devices’ data only (*t*(1016) = -6.21, *p* < .001, *d* = 0.41, 95% CI [-0.63, -0.32]; S1:
*M* = 0.14D, *SD* = 0.99D; S2: *M* = -0.33D, *SD* = 1.23D).

In the younger sample (S1, *N* = 339), the regression model with the predictors grade and gender did not reach statistical significance (*R^2^* = .001, *F*(2, 336) = 0.20, *p* = .822). In the “all possible subsets” approach, the best-fitting model did not include any of the given predictors. From the models including predictors, the best-fitting model included grade as only predictor, and it did also not reach statistical significance (*R^2^* = .001, *F*(1, 337) = 0.390, *p* = .533).

In the older sample (S2, *N* = 668), both grade and gender were identified as significant predictors of SER (see Supplementary Table 19, model A), with the respective regression model being overall significant (*R^2^* = .032, *F*(3, 664) = 7.27, *p* < .001). This model was also identified as the most promising one via the “all possible subsets” approach. The regression model including grade, gender and grade × gender as predictors (model B) exhibited a slightly higher adjusted R^2^ and was thus also fitted, despite a substantial BIC difference to model A. Furthermore, and other than in the manuscript’s data, the model with age, gender, grade and age × gender as predictors performed (almost) equally in the “all possible subsets” approach as model B for these data – though fitting a model with both age and grade included may presumably be problematic regarding multicollinearity, since these two variables are highly correlated S2 (Spearman’s rho; *r*_s_ = .76). Model B also explained variance in SER (*R^2^* = .033, *F*(5, 662) = 4.50, *p* < .001), but while grade 10 (*B* = -0.58, *p* = .001) significantly predicted SER in this model, the other predictors did not (grade 9: *B* = -1.91, *p* = .057; grade (9) × gender: *B* = 0.18, *p* = .445; grade (10) × gender: *B* = 0.17, *p* = .465; unlike here, grade 9 also significantly predicted SER in model B in the manuscript’s data). An F-test for nested models showed that model B did not fit the data better than model A (*F*(2, 664) = 0.37, *p* = .689).

SER was more myopic in females than males in S2 (females: *M* = -0.45D, *SD* = 1.31D, males:
*M* = -0.25D, *SD* = 1.15D). Post-hoc Holm-corrected Welch two sample t-tests showed that the SER of grade 9 (*M* = -0.33D, *SD* = 1.21D) and grade 10 (*M* = -0.58D, *SD* = 1.40D) participants was significantly more myopic than that of grade 8 participants (*M* = -0.10D, *SD* = 1.00D; grade 8 vs. 9: *t*(436.8) = 2.25, *p* = .049, *d* = 0.21, 95% CI [0.03, 0.44]; grade 8 vs. 10: *t*(388.14) = 4.16, *p* < .001,
*d* = 0.40, 95% CI [0.25, 0.71]). Furthermore, and other than in the manuscript’s data, the SER of grade 10 participants was significantly more myopic than that of grade 9 participants
(*t*(425.81) = 1.99, *p* = .049, *d* = 0.19, 95% CI [0.00, 0.49]).

Supplementary Table 16

*Mean SER and Standard Error per Gender by Grade for the Complete Data Without Linear Transformation of the A09 Device Data*

| **sample** | **female** | **male** | **all genders** |
| --- | --- | --- | --- |
| S1 |  |  |  |
| grade 3 | 0.39D (0.10) | 0.17D (0.09) | 0.27D (0.07) |
| grade 4 | 0.03D (0.11) | 0.17D (0.10) | 0.11D (0.08) |
| S2 |  |  |  |
| grade 8 | -0.06D (0.08) | -0.01D (0.08) | -0.03D (0.06) |
| grade 9 | -0.37D (0.09) | -0.27D (0.10) | -0.32D (0.07) |
| grade 10 | -0.69D (0.12) | -0.26D (0.09) | -0.45D (0.07) |

*Note.* The data for all genders include eight more participants than the data of males and females combined due to four non-binary participants and four participants with unknown gender. The corresponding data are presented in Figure 3 in the manuscript. Here, the SER is slightly less myopic than in the manuscript’s data, though the general tendencies are comparable.

Supplementary Table 17

*Mean SER and Standard Error per Gender by Grade for the A12R Devices’ Data Only*

| **sample** | **female** | **male** | **all genders** |
| --- | --- | --- | --- |
| S1 |  |  |  |
| grade 3 | 0.31D (0.12) | 0.07D (0.10) | 0.17D (0.08) |
| grade 4 | -0.01D (0.13) | 0.21D (0.08) | 0.11D (0.08) |
| S2 |  |  |  |
| grade 8 | -0.14D (0.11) | -0.06D (0.08) | -0.10D (0.07) |
| grade 9 | -0.47D (0.12) | -0.22D (0.11) | -0.33D (0.08) |
| grade 10 | -0.72D (0.16) | -0.47D (0.12) | -0.58D (0.10) |

*Note.* The data for all genders include four more participants than the data of males and females combined due to two non-binary participants and two participants with unknown gender. The corresponding data are presented in Figure 3 in the manuscript. Here, other than in the manuscript’s data, male participants of grade 3 have a more myopic SER than male participants of grade 4, and the SER difference between male participants of grade 9 & 10 is somewhat larger than in the manuscript’s data, due to the grade 9 prevalence being less, and the grade 10 prevalence being more myopic than in the manuscript’s data. Other than that, the general tendencies are comparable.

Supplementary Table 18

Coefficient Estimates of Multiple Linear Regression Model A for S2 for the Complete Data Without Linear Transformation of the A09 Device Data

| **coefficient** | ***B*** | **95% CI** | **SE** | ***t*** | ***p*** |
| --- | --- | --- | --- | --- | --- |
| intercept | -0.14 | [-0.30, 0.02] | 0.08 | -1.74 | .083 |
| grade (9) | -0.27 | [-0.46, -0.09] | 0.09 | -2.91 | .004 |
| grade (10) | -0.41 | [-0.60, -0.22] | 0.10 | -4.30 | < .001 |
| gender | 0.18 | [0.03, 0.34] | 0.08 | 2.35 | .020 |

*Note.* CI = confidence interval, SE = standard error. The corresponding data are presented in Table 4 in the manuscript.

Supplementary Table 19

Coefficient Estimates of Multiple Linear Regression Model A for S2 for the A12R Devices’ Data Only

| **coefficient** | ***B*** | **95% CI** | **SE** | ***t*** | ***p*** |
| --- | --- | --- | --- | --- | --- |
| intercept | -0.21 | [-0.40, -0.02] | 0.10 | -2.13 | .033 |
| grade (9) | -0.23 | [-0.45, -0.01] | 0.11 | -2.02 | .044 |
| grade (10) | -0.48 | [-0.71, -0.25] | 0.12 | -4.16 | < .001 |
| gender | 0.19 | [0.01, 0.38] | 0.09 | 2.06 | .040 |

*Note.* CI = confidence interval, SE = standard error. The corresponding data are presented in Table 4 in the manuscript. Here, other than in the manuscript’s data, grade 9 is not a significant predictor in model B.

Supplementary Table 20

Prevalence of Uncorrected Myopia in S1 and S2 Overall and by Grade for the Complete Data Without Linear Transformation of the A09 Device Data

| **sample** | **myopia cut-off SER ≤ -0.75D** | | **myopia cut-off SER ≤ -1D** | |
| --- | --- | --- | --- | --- |
|  | ***N*** | **% uncorrected** | ***N*** | **% uncorrected** |
| S1 | 41 | 51.2 | 39 | 48.7 |
| grade 3 | 20 | 55.0 | 18 | 50.0 |
| grade 4 | 21 | 47.6 | 21 | 47.6 |
| S2 | 190 | 40.5 | 154 | 31.8 |
| grade 8 | 35 | 42.9 | 27 | 33.3 |
| grade 9 | 73 | 37.0 | 59 | 30.5 |
| grade 10 | 82 | 42.7 | 68 | 32.4 |

*Note.* *N* indicates the number of myopic participants per the respective cut-off. The given prevalence indicates the percentage of myopic participants without visual aid based on all myopic participants. The corresponding data are presented in Table 5 in the manuscript. Here, the prevalence of uncorrected myopia for grade 8 is somewhat lower than in the manuscript’s data, but the general tendencies are similar.

Supplementary Table 21

Prevalence of Uncorrected Myopia in S1 and S2 Overall and by Grade for the A12R Devices’ Data Only

| **sample** | **myopia cut-off SER ≤ -0.75D** | | **myopia cut-off SER ≤ -1D** | |
| --- | --- | --- | --- | --- |
|  | ***N*** | **% uncorrected** | ***N*** | **% uncorrected** |
| S1 | 28 | 57.1 | 26 | 53.8 |
| grade 3 | 13 | 61.5 | 11 | 54.5 |
| grade 4 | 15 | 53.3 | 15 | 53.3 |
| S2 | 141 | 43.3 | 110 | 32.7 |
| grade 8 | 26 | 42.3 | 20 | 35.0 |
| grade 9 | 52 | 44.2 | 40 | 35.0 |
| grade 10 | 63 | 42.9 | 50 | 30.0 |

*Note. N* indicates the number of myopic participants per the respective cut-off. The given prevalence indicates the percentage of myopic participants without visual aid based on all myopic participants. The corresponding data are presented in Table 5 in the manuscript. Here, the prevalence of uncorrected myopia for S1 as well as grade 9 is somewhat higher than in the manuscript’s data, while that of grade 8 for the ≤ -0.75D cut-off is somewhat lower. However, the general tendencies are comparable.

Supplementary Table 22

*Prevalence and Standard Error of Corrected and Uncorrected Myopia by Grade Relative to the Overall Sample for the Complete Data Without Linear Transformation of the A09 Device Data*

| **sample** | **myopia cut-off SER ≤ -0.75D** | |
| --- | --- | --- |
|  | **% uncorrected (SE)** | **% corrected (SE)** |
| S1 |  |  |
| grade 3 | 4.5 (1.3) | 3.7 (1.2) |
| grade 4 | 4.1 (1.3) | 4.5 (1.3) |
| S2 |  |  |
| grade 8 | 4.3 (1.1) | 5.8 (1.3) |
| grade 9 | 7.7 (1.4) | 13.2 (1.8) |
| grade 10 | 10.4 (1.7) | 14.0 (1.9) |

*Note.* SE = standard error. The corresponding data are presented in Figure 4 in the manuscript.

Supplementary Table 23

*Prevalence and Standard Error of Corrected and Uncorrected Myopia by Grade Relative to the Overall Sample for the A12R Devices’ Data Only*

| **sample** | **myopia cut-off SER ≤ -0.75D** | |
| --- | --- | --- |
|  | **% uncorrected (SE)** | **% corrected (SE)** |
| S1 |  |  |
| grade 3 | 4.8 (1.7) | 3.0 (1.3) |
| grade 4 | 4.5 (1.6) | 4.0 (1.5) |
| S2 |  |  |
| grade 8 | 4.9 (1.4) | 6.7 (1.7) |
| grade 9 | 10.0 (2.0) | 12.6 (2.2) |
| grade 10 | 12.2 (2.2) | 16.2 (2.5) |

*Note.* SE = standard error. The corresponding data are presented in Figure 4 in the manuscript.

## Supplementary Information S3: Further Uncorrected Myopia Data for the Complete Data Without Linear Transformation of the A09 Device Data

The prevalence of uncorrected myopia for the complete data without linear transformation of the A09 device data was 55.0% for males and 47.6% for females in the younger sample (S1), and 42.5% for males and 39.3% for females in the older sample (S2). With the SER ≤ -1D myopia cut-off, these numbers were 52.6% (28.6%) for males and 45.0% (34.1%) for females in S1 (S2).

Of the myopic participants in the younger sample (S1), 38.9% (7 of 18 children) in the three schools with the lowest social index levels – i.e., lower social burden –, and 60.9% (14 of 23 children) in the three schools with the highest social index levels had uncorrected myopia. It is, however, important to consider that data on (un)corrected myopia in S1 is based on 41 myopic participants only.

## Supplementary Information S4: Further Uncorrected Myopia Data for the A12R Devices’ Data Only

The prevalence of uncorrected myopia for the A12R devices’ data only was 66.7% for males (this being somewhat higher than in the manuscript’s data) and 50.0% for females in the younger sample (S1), and 44.3% for males and 43.0% for females in the older sample (S2). With the SER ≤ -1D myopia cut-off, these numbers were 63.6% (28.3%) for males and 46.7% (36.5%) for females in S1 (S2), with the prevalence for males in S1 again somewhat higher than in the manuscript’s data.

Of the myopic participants in the younger sample (S1), 45.5% (5 of 11 children) in the three schools with the lowest social index levels – i.e., lower social burden –, and 64.7% (11 of 17 children) in the three schools with the highest social index levels had uncorrected myopia. It is, however, important to consider that data on (un)corrected myopia in S1 is based on 28 myopic participants only.

# Detailed Statistical Parameters

*In the following, we present the statistical parameters of the analyses we performed with regard to SER associations that are not reported on in detail in the manuscript.*

## Supplementary Information S5: Detailed Statistical Parameters of the Calculations Regarding SER Associations

SER was significantly more myopic in S2 (*N* = 1029) than S1 (*N* = 486; *t*(1513) = -7.17, *p* < .001,
*d* = 0.39, 95% CI [-0.58, -0.33]; S1: *M* = 0.08D, *SD* = 1.06D; S2: *M* = -0.37D, *SD* = 1.20D).

For S1, the regression model with the predictors grade and gender did not reach statistical significance (*R^2^* = .004, *F*(2, 480) = 0.99, *p* = .371). The model with only grade as predictor did also not reach statistical significance (*R^2^* = .004, *F*(1, 481) = 1.84, *p* = .175).

For S2, the regression model with grade and gender as predictors (model A) was overall significant (*R^2^* = .025, *F*(3, 1011) = 8.59, *p* < .001), as were both predictors (grade 9: *B* = -0.26, *p* = .005; grade 10: *B* = -0.40, *p* < .001; gender: *B* = 0.18, *p* = .015). The regression model with grade, gender, and grade × gender as predictors (model B) was overall significant (*R^2^* = .029, *F*(5, 1009) = 5.96,
*p* < .001), but only grade was a significant predictor (grade 9: *B* = *-0.31*, *p* = .023; grade 10:
*B* = -0.60, *p* = < .001), and gender as well as both gender × grade terms were not significant (gender: *B* = 0.04, *p* = .750; grade (9) × gender: *B* = 0.08, *p* = .643; grade (10) × gender: *B* = 0.35, *p* = .057). An F-test for nested models (*F*(2, 1011) = 1.97, *p* = .139) showed that model B did not fit the data better than model A.

Post-hoc, Holm-corrected Welch two-sample t-tests showed that the SER of grade 9 and grade 10 participants was significantly more myopic than that of grade 8 participants (grade 8 vs. 9:
*t*(661.23) = 3.09, *p* = .004, *d* = 0.24, 95% CI [0.10, 0.44]; grade 8 vs. 10: *t*(615.36) = 4.42, *p* < .001,
*d* = 0.34, 95% CI [0.22, 0.58]). The SER was not significantly different between grade 9 and 10 participants (*t*(662.08) = 1.33, *p* = .183, *d* = 0.10, 95% CI [-0.06, 0.32]).

# Results of the “All Possible Subsets” Analyses

*In the following, we present the results of the “all possible subsets” analyses on regression models for the SER data.*

Supplementary Table 24

*“All Possible Subsets” Regression Output for the Younger Sample (S1)*

| **intercept** | **age** | **gender** | **grade** | **age × gender** | **age × grade** | **gender × grade** | **age × gender × grade** | **adjusted R^2^** | **BIC** | **delta** |
| --- | --- | --- | --- | --- | --- | --- | --- | --- | --- | --- |
| 0.082 | NA | NA | NA | NA | NA | NA | NA | 0.000 | 1441.756 | 0.000 |
| 0.147 | NA | NA | + | NA | NA | NA | NA | 0.004 | 1446.090 | 4.334 |
| 0.102 | NA | + | NA | NA | NA | NA | NA | 0.000 | 1447.789 | 6.034 |
| 0.254 | 0.000 | NA | NA | NA | NA | NA | NA | 0.000 | 1447.848 | 6.092 |
| -0.270 | 0.000 | NA | + | NA | NA | NA | NA | 0.005 | 1451.893 | 10.137 |
| 0.167 | NA | + | + | NA | NA | NA | NA | 0.004 | 1452.121 | 10.365 |
| 0.266 | 0.000 | + | NA | NA | NA | NA | NA | 0.000 | 1453.889 | 12.133 |
| 0.254 | NA | + | + | NA | NA | + | NA | 0.010 | 1455.580 | 13.824 |
| 1.478 | 0.000 | + | NA | + | NA | NA | NA | 0.008 | 1456.540 | 14.784 |
| 0.263 | 0.000 | NA | + | NA | + | NA | NA | 0.007 | 1456.899 | 15.143 |
| -0.260 | 0.000 | + | + | NA | NA | NA | NA | 0.005 | 1457.903 | 16.147 |
| 0.931 | 0.000 | + | + | + | NA | NA | NA | 0.012 | 1461.028 | 19.272 |
| -0.267 | 0.000 | + | + | NA | NA | + | NA | 0.012 | 1461.160 | 19.404 |
| 0.259 | 0.000 | + | + | NA | + | NA | NA | 0.008 | 1462.969 | 21.213 |
| 1.524 | 0.000 | + | + | + | + | NA | NA | 0.015 | 1465.910 | 24.154 |
| 0.237 | 0.000 | + | + | NA | + | + | NA | 0.014 | 1466.279 | 24.523 |
| 0.501 | 0.000 | + | + | + | NA | + | NA | 0.013 | 1466.545 | 24.789 |
| 1.105 | 0.000 | + | + | + | + | + | NA | 0.016 | 1471.524 | 29.768 |
| 1.101 | 0.000 | + | + | + | + | + | + | 0.016 | 1477.704 | 35.948 |

*Note.* Some values in the column “age” are zero due to rounding – they are not actually zero. “Delta” refers to the difference in BIC.

Supplementary Table 25

*“All Possible Subsets” Regression Output for the Older Sample (S2)*

| **intercept** | **age** | **gender** | **grade** | **age × gender** | **age × grade** | **gender × grade** | **age × gender × grade** | **adjusted R^2^** | **BIC** | **delta** |
| --- | --- | --- | --- | --- | --- | --- | --- | --- | --- | --- |
| -0.153 | NA | NA | + | NA | NA | NA | NA | 0.020 | 3254.179 | 0.000 |
| -0.260 | NA | + | + | NA | NA | NA | NA | 0.026 | 3255.134 | 0.955 |
| 1.104 | 0.000 | + | NA | NA | NA | NA | NA | 0.017 | 3257.307 | 3.129 |
| 1.124 | 0.000 | NA | NA | NA | NA | NA | NA | 0.009 | 3257.949 | 3.770 |
| 2.403 | -0.001 | + | NA | + | NA | NA | NA | 0.021 | 3259.900 | 5.722 |
| -0.372 | NA | NA | NA | NA | NA | NA | NA | 0.000 | 3259.919 | 5.740 |
| -0.478 | NA | + | NA | NA | NA | NA | NA | 0.007 | 3260.473 | 6.295 |
| -0.334 | 0.000 | NA | + | NA | NA | NA | NA | 0.020 | 3261.030 | 6.851 |
| -0.246 | 0.000 | + | + | NA | NA | NA | NA | 0.026 | 3262.056 | 7.877 |
| -0.177 | NA | + | + | NA | NA | + | NA | 0.030 | 3265.015 | 10.836 |
| 0.980 | 0.000 | + | + | + | NA | NA | NA | 0.029 | 3265.577 | 11.398 |
| -0.180 | 0.000 | + | + | NA | NA | + | NA | 0.030 | 3271.938 | 17.759 |
| -1.280 | 0.000 | NA | + | NA | + | NA | NA | 0.021 | 3273.818 | 19.639 |
| -1.189 | 0.000 | + | + | NA | + | NA | NA | 0.027 | 3274.837 | 20.659 |
| 0.448 | 0.000 | + | + | + | NA | + | NA | 0.030 | 3278.417 | 24.238 |
| 0.078 | 0.000 | + | + | + | + | NA | NA | 0.030 | 3278.525 | 24.346 |
| -1.267 | 0.000 | + | + | NA | + | + | NA | 0.031 | 3284.409 | 30.231 |
| -0.742 | 0.000 | + | + | + | + | + | NA | 0.031 | 3291.115 | 36.936 |
| -1.956 | 0.000 | + | + | + | + | + | + | 0.032 | 3304.360 | 50.181 |

*Note.* Some values in the column “age” are zero due to rounding – they are not actually zero. “Delta” refers to the difference in BIC.
